# Exo‐ and endophytic fungi enable rapid transfer of nutrients from ant waste to orchid tissue

**DOI:** 10.1111/nph.18761

**Published:** 2023-02-17

**Authors:** Christian Gegenbauer, Anke Bellaire, Arno Schintlmeister, Markus C. Schmid, Markus Kubicek, Hermann Voglmayr, Gerhard Zotz, Andreas Richter, Veronika E. Mayer

**Affiliations:** ^1^ Division of Structural and Functional Botany, Department of Botany and Biodiversity Research University of Vienna Rennweg 14 1030 Wien Austria; ^2^ Division of Terrestrial Ecosystem Research, Centre for Microbiology and Ecosystem Science University of Vienna Djerassiplatz 1 1030 Wien Austria; ^3^ Division of Microbial Ecology and Large‐Instrument Facility of Environmental and Isotope Mass Spectrometry, Centre for Microbiology and Environmental Systems Science University of Vienna Djerassiplatz 1 1030 Vienna Austria; ^4^ Institute of Chemical Technologies and Analytics, TU Wien Getreidemarkt 9/164 1060 Vienna Austria; ^5^ Mycology Research Group, Department of Botany and Biodiversity Research University of Vienna Rennweg 14 1030 Wien Austria; ^6^ Institute of Forest Entomology, Forest Pathology and Forest Protection University of Natural Resources and Life Sciences, Vienna (BOKU) Peter‐Jordan‐Strasse 82 1190 Wien Austria; ^7^ Institute for Biology and Environmental Sciences Carl von Ossietzky University Oldenburg Oldenburg Germany; ^8^ Smithsonian Tropical Research Institute Apdo 2072 Balboa Panama

**Keywords:** ^15^N uptake, *Caularthron bilamellatum*, endophytes, myrmecophytes, NanoSIMS, stable isotope tracing, ToF‐SIMS

## Abstract

- The epiphytic orchid *Caularthron bilamellatum* sacrifices its water storage tissue for nutrients from the waste of ants lodging inside its hollow pseudobulb. Here, we investigate whether fungi are involved in the rapid translocation of nutrients.
- Uptake was analysed with a ^15^N labelling experiment, subsequent isotope ratio mass spectrometry (IRMS) and secondary ion mass spectrometry (ToF‐SIMS and NanoSIMS).
- We encountered two hyphae types: a thick melanized type assigned to ‘black fungi’ (Chaetothyriales, Cladosporiales, and Mycosphaerellales) in ant waste, and a thin endophytic type belonging to Hypocreales. In few cell layers, both hyphae types co‐occurred. ^15^N accumulation in both hyphae types was conspicuous, while for translocation to the vessels only Hypocreales were involved. There is evidence that the occurrence of the two hyphae types results in a synergism in terms of nutrient uptake.
- Our study provides the first evidence that a pseudobulb (=stem)‐born endophytic network of Hypocreales is involved in the rapid translocation of nitrogen from insect‐derived waste to the vegetative and reproductive tissue of the host orchid. For *C. bilamellatum* that has no contact with the soil, ant waste in the hollow pseudobulbs serves as equivalent to soil in terms of nutrient sources.

## Abstract 2

See also the Commentary on this article by Leroy, **238**: 1752–1754.

## Introduction

The canopy of tropical forests is a challenging habitat with often harsh environmental conditions. High radiation and limited availability of water and nutrients force organisms to develop a multitude of strategies to grow and reproduce (Nakamura *et al*., 2017). This is especially true for epiphytes which, unlike terrestrial plants, lack direct soil contact. To be able to meet their nutritional needs, vascular epiphytes have evolved remarkable morphological adaptations: for example, aerial roots with a velamen radicum, leaf‐absorbing trichomes, leaf arrangements for litter‐trapping, adventitious roots or leaves forming tanks with water and nutrient‐storing phytotelmata (Benzing, 1970, 2000; Zotz & Hietz, 2001; Zotz & Richter, 2006; Zotz *et al*., 2021). Such adaptations enable vascular epiphytes to take up nutrients from clouds, rainfall, throughfall and stemflow water and/or from decomposing organic matter from plants, insects, birds or other organisms (Benzing, 1990; Winkler & Zotz, 2010; Zotz & Winkler, 2013; Gotsch *et al*., 2015; Leroy *et al*., 2017; Sun *et al*., 2020). Epiphytes also frequently engage in mutualistic relationships with other organisms to escape nutrient scarcity, for example with mycorrhizal fungi (Rains *et al*., 2003; Looby *et al*., 2020), or with ants.

Specific and obligate mutualisms between ants and plants with distinct morphological plant structures that serve as nesting space for ants (domatia) occur only in a relatively small number of plant species (myrmecophytes), and epiphytes account for a significant proportion (Chomicki & Renner, 2015). An important reason for the latter might be that the association with ants enables access to nutrient resources. Irrespective of a mutualistic relationship with plants, ant nests and nest environments are hotspots of nutrients (C, N, P and K) and cations (Al, Ca, Mg and Na). A key role plays ant waste generated from plant material, faeces, carcasses and metabolic products typical for ants (Beattie, 1985; Blüthgen *et al*., 2001; Farji‐Brener & Werenkraut, 2017). In myrmecophytes, ants accumulate such organic waste in the nesting sites inside the host plant (Treseder *et al*., 1995; Fischer *et al*., 2003; Solano & Dejean, 2004; Defossez *et al*., 2011; Bazile *et al*., 2012; Lucas *et al*., 2018). The transfer of nutrients from such ant waste to plants has been demonstrated in ground‐rooted myrmecophytes (Fischer *et al*., 2003; Solano & Dejean, 2004; Defossez *et al*., 2011; Bazile *et al*., 2012) as well as in myrmecophytic epiphyte genera like *Dischidia* sp. (Apocynaceae) with its domatia from folded leaves (Treseder *et al*., 1995), in *Lecanopteris* sp. (Polypodiaceae) with its hollow rhizomes (Gay, 1993), or *Hydnophytum*, *Myrmecodia* and *Squamellaria* sp. (Rubiaceae), which exhibit a prominent caudex with natural cavities (Huxley, 1978; Rickson, 1979; Chomicki & Renner, 2019). The importance of nutrient supply from ants to plants may be different depending on ecology and habitat of the host plant, suggesting that epiphytes with missing soil connection and limited access to mineral nutrients benefit to a much higher degree than ground‐rooted plants (Janzen, 1974; Rico Gray, 1987; Gay, 1993; Treseder *et al*., 1995; Gegenbauer *et al*., 2012).

Remarkably, for some epiphytes, access to nutrients may even be more important than water storage tissue. This is the case in species of two myrmecophytic orchid genera (*Myrmecophila* and *Caularthron*) which sacrifice a considerable part of water storage tissue to form hollow pseudobulbs that are used by ants as nesting space even though water is the most limiting resource for growth and survival of epiphytes (Zotz *et al*., 2021). In case of *Caularthron*, up to 50% of pseudobulb tissue is lost during pseudobulb maturation (G. Zotz, unpublished; Gegenbauer *et al*., 2012), which raises the question of a trade‐off between water and nutrients. Indeed, carbon uptake from the debris of ants placed in pseudobulbs has been demonstrated for *Myrmecophila tibicinis* (Orchidaceae; Rico Gray *et al*., 1989) and nitrogen transfer from ants to plants in *Caularthron bilamellatum* (Orchidaceae; Gegenbauer *et al*., 2012). In the latter, a multitude of organic and inorganic nitrogen sources were taken up by hollow pseudobulbs under field conditions and even translocated into seeds. Enzyme kinetics suggested the presence of an active uptake system but when investigating the morphology and anatomy of the pseudobulbs highly specialized surface structures as known from epiphytic Rubiaceae living with ants (Janzen, 1974; Huxley, 1978; Rickson, 1979), were not found. Instead, we frequently observed a conspicuous colonization by fungal hyphae in the ant debris as well as in the pseudobulb tissue close to the cavity (fig. 2D in Gegenbauer *et al*., 2012). This is prompting us to suggest that these fungi may facilitate plant nutrient uptake from ant waste as it is well known from root – fungi associations. Usually, ant‐associated fungi are restricted to ant waste and dead cells and do not penetrate living plant cells (Defossez *et al*., 2009; Voglmayr *et al*., 2011; Vasse *et al*., 2017; Mayer *et al*., 2018).

Endophytic fungi, which may inhabit any living plant tissue, are frequently encountered throughout the plant kingdom (Carroll, 1988; Rasmussen, 2002; McCormick *et al*., 2004; Bayman & Otero, 2006; Suárez *et al*., 2006; Dearnaley, 2007; Yuan *et al*., 2009). They can involve commensals, latent pathogens (Carroll, 1988; Redman *et al*., 2001) as well as plant‐beneficial fungi, which may contribute to plant fitness through secondary metabolites, for example antimicrobial substances (Vaz *et al*., 2009; Ratnaweera & de Silva, 2017) or plant hormones (Salazar‐Cerezo *et al*., 2018). However, to our best knowledge, nothing is yet known about nonmycorrhizal endophytic fungi involved in nutrient transfer in the tissue of pseudobulbs of epiphytic orchids.

In the present study, we aimed at investigating whether the endophytic fungi in the tissue of pseudobulbs play a crucial role in the mutualistic association between the epiphytic orchid *Caularthron bilamellatum* and its inhabiting ants. We hypothesize that the endophytic fungi are the ‘active uptake system’ mediating the rapid translocation of nutrients from the ant waste in the cavity of the pseudobulb into the vegetative and reproductive plant tissue.

To address this aim, we conducted a labelling experiment on *C. bilamellatum* pseudobulbs under field conditions using ^15^N stable isotope tracing for a period of 1, 2, 4 and 8 d. Mature pseudobulbs from plants inhabited by different ant species were labelled by placing a solid organic ^15^N source into the hollow chamber. Ants were barred from entering and spreading the label. ^15^N uptake into plant tissue was then analysed using isotope ratio mass spectrometry (IRMS), as well as secondary ion mass spectrometry (ToF‐ and NanoSIMS). Further, the tissue was screened for fungal endophytes using light microscopy (LM), scanning electron microscopy (SEM) and X‐ray micro‐computed tomography (micro‐CT). Fungi were isolated from ant waste and plant tissue, cultured and sequenced for identification. We aimed at correlating fungal morphology in LM with secondary ion mass spectrometry images and to trace ^15^N uptake from the pseudobulb cavity to vessels in the plant tissue. Finally, we tested whether ant presence enhances nutrient uptake capabilities by comparing ant‐inhabited pseudobulbs rich in detritus and fungi with closed pseudobulbs free of ants.

## Materials and Methods

### Sample collection and experimental set‐up

**Fig. 1:**
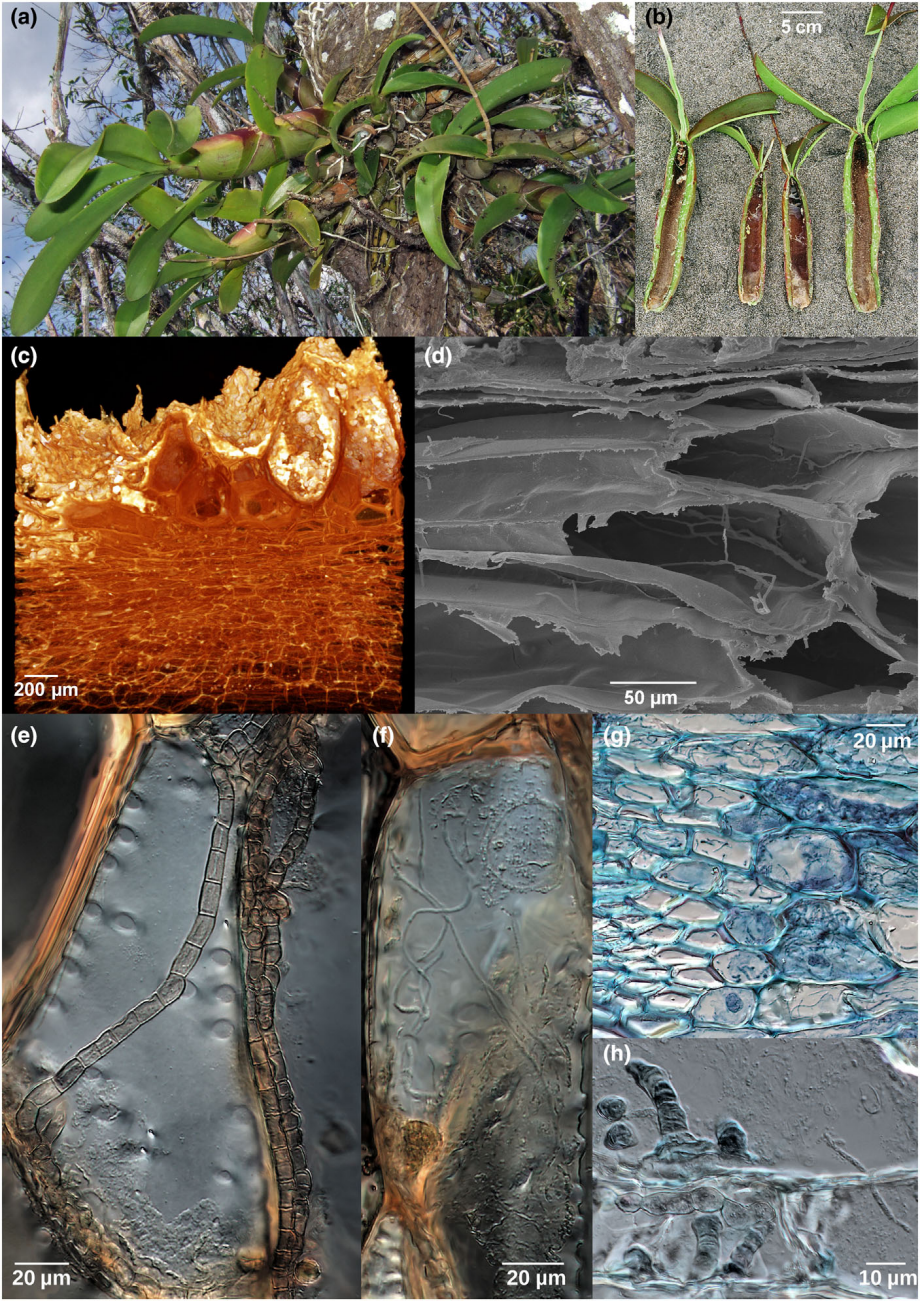
Morphology of *Caularthron bilamellatum* (Orchidaceae), anatomy of pseudobulbs inhabited by *Azteca* cf *velox* and endophytic fungi. (a) Epiphytic growth of *C. bilamellatum* with ants inhabiting the hollow pseudobulbs (pbs). (b) Longitudinal section through pbs displaying the hollow chamber, which forms upon maturation. Ants can enter through a basal slit (bottom). (c) Micro‐CT image of transversal section of pb. Big cells of the former water storage tissue are torn open and filled with ant waste (white). (d) Scanning electron microscopy image of living plant tissue several cell layers outward from the pb cavity. Thin fungal hyphae are growing along and across cell walls. (e) Thick, distinctly septate melanized fungal hyphae (black fungi) growing in and through dead cells adjacent to the pseudobulb cavity. Note abundant pits. (f) Thin hyaline string‐like hyphae (Hypocreales) growing in living cells several layers outward from the pb cavity. The cell nucleus can be seen in the upper right corner. (g) Thin hyphae (visible as blue strings) growing deeper into living tissue and towards the vessels (bottom left). (h) Interface *c*. 2–4 cell layers outward from the pb cavity with both thick (black fungi, middle left) and thin (Hypocreales, middle right) hyphae within living cells, often crossing cell walls.

We collected 15 mature, hollow pseudobulbs of *Caularthron bilamellatum* (Rchb.f.) R.E.Schult. (Orchidaceae) growing on *Annona glabra* (Annonaceae) trees (Fig. 1a) at each of six different sampling plots along the southern shore of Barro Colorado Island (BCI), Republic of Panama. A sampling plot was defined as an isolated group of trees inhabited by a single ant species. In total, 90 pseudobulbs were taken for the labelling experiment. In rare cases, the formation of an opening slit during maturation fails and the pseudobulb remains closed and inaccessible to ants and microorganisms. Two such samples could be obtained to investigate nutrient uptake in uninhabited pseudobulbs. The experimental set‐up took place at the laboratories of the Smithsonian Tropical Research Station (STRI).

#### ^15^N agar disc preparation

A medium containing 10 mg ml^−1^ Agar and 10 mg ml^−1 15^N isotopically labelled algal amino acid mixture (98 atom% ^15^N; Sigma Aldrich) was poured into a Petri dish. Discs of similar size with a diameter of 1 cm were cut using a hollow drill sampling tool.

#### Pseudobulb incubation

Upon maturation, pseudobulbs of *C. bilamellatum* desiccate, forming a hollow chamber (Fig. 1b, Supporting Information Fig. S1) and a slit at the base allowing ants to access the cavity. After the natural opening at the base of the pseudobulbs was enlarged, the remaining ants were manually removed and the agar disc containing the high molecular weight ^15^N label was placed on the ant debris in the apical region of the pseudobulb cavity. The opening was then sealed with parafilm leaving a small slit mimicking the original state to maintain the natural micro‐climate within the cavity. For each plot, two pseudobulbs per time point (1, 2, 4 and 8 d) were incubated under field conditions but kept free of ants (= 8 per plot), in total 48 pseudobulbs. Table S1 gives the sample size per sampling plot.

Upon reaching the intended incubation time, pseudobulbs were cut open, the remains of the agar discs removed and rinsed twice with 10 mM CaCl_2_ solution and distilled water to remove label adhering to the surface of the pseudobulb cavity. Several small samples of each pseudobulb's apical region were cut out and either fixed in 70% EtOH for sectioning or dried for 24 h at 60°C for IRMS analysis. Also, leaf tips *c*. 10–15 cm distant from the labelling site were harvested and dried. Unfortunately, some samples were lost during shipping to Vienna leaving 40 pseudobulb and 37 leaf samples for IRMS.

### Morpho‐anatomical investigation

For microtome sectioning, the samples fixed in EtOH were dehydrated and embedded in a resin based on 2‐hydroxyethyl methacrylate (Technovit 7100; Heraeus Kulzer GmbH, Germany) according to Igersheim (1993) and Igersheim & Cichocki (1996), cut into 5–10‐μm‐thick sections with a Leitz 1515 rotary microtome (Leica Microsystems AG, Wetzlar, Germany) and counterstained with Ruthenium Red against Toluidine Blue.

Scanning electron microscopy (SEM) was performed on a Jeol JSM‐6390 (Jeol USA Inc., Peabody, MA, USA) as described in Gegenbauer *et al*. (2012).

For a 3D reconstruction of the waste distribution, apical pseudobulb parts were analysed with a microXCT‐200 X‐ray tomography (micro‐CT) system (Zeiss Microscopy, Jena, Germany). Sample preparation and reconstruction were performed according to Staedler *et al*. (2013).

### Isotope ratio mass spectrometry

The ^15^N : ^14^N isotopic ratios were determined using an elemental analyser (EA 110; CE Instruments, Milan, Italy) coupled to an isotope ratio mass spectrometer (Delta Plus; Finnigan MAT, Bremen, Germany) as described previously (Gegenbauer *et al*., 2012).

Nitrogen isotope ratio deviation (δ) values were calculated as:

$$\delta^{15}N\;\left(‰\right)=\left(R_{\text{sample}}/R_{\text{standard}}-1\right)\times 1000$$

*R*_sample_ and *R*_standard_ are the molar ratios of ^15^N : ^14^N of the sample and the standard (atmospheric air; Peterson & Fry, 1987).

The measured ^15^N enrichment per sample was calculated to atom% excess as (APE):

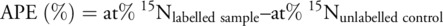




### Secondary ion mass spectrometry (ToF‐SIMS and NanoSIMS)

Four 70% EtOH fixed samples of pseudobulbs inhabited by *Azteca* cf *velox*, one labelled for 24 h, one for 8 d and two unlabelled controls were embedded in an epoxy resin (Araldite 502; EMS, Hatfield, PA, USA) and sectioned with a Reichert‐Jung UltraCut E Ultramicrotome using glass knives into 0.5–1‐μm semithin sections. Sections were deposited either onto antimony‐doped silicon wafer platelets (7.1 × 7.1 × 0.75 mm; Active Business Co., Brunnthal, Germany) or indium tin oxide (ITO)‐coated glass slides (7.1 × 7.1 × 1.1 mm; Praezisions Glas & Optik GmbH, Iserlohn, Germany) and kept at 80°C for 1 h. For NanoSIMS analysis, sections were sputter‐coated with an AuPd (80/20) layer of 30 nm (nominal thickness) to prevent electric charging through the NanoSIMS measurement process. For ToF‐SIMS, the sections remained uncoated.

#### ToF‐SIMS

At the Institute of Chemical Technologies and Analytics of the Technical University of Vienna, a TOF‐SIMS 5 instrument (Ion‐Tof, Münster, Germany), utilizing 25 keV Bi^+^ as primary ions in imaging mode was used to analyse negative secondary ions. The ion beam was chopped into eight short pulses (burst mode), which was necessary to attempt discrimination of several ion species at the nominal masses 26 and 27. Areas of 300 × 300 μm^2^ were scanned using a raster of 2048 × 2048 pixels. Before measurement, an area of 900 × 900 μm^2^ was presputtered with 1 keV Cs^+^ ions to remove *c*. 300 nm from the specimen surface that was altered from exposure to air (measurable as a change of secondary ion yield with depth). During measurement, the same parameters were used to remove *c*. 1 nm after each *x*–*y* image (i.e. 2048 × 2048 mass spectra). An electron flood gun (20 eV) was used for charge compensation.

To determine the local nitrogen isotope composition, the secondary ion counts of ^12^C^14^N^−^ and ^12^C^15^N^−^ of all eight bursts were integrated and used for calculation as their signal intensities were considerably stronger than those of ^14^N^−^ and ^15^N^−^. It should be noted that the mass resolving power achieved in the burst mode (*M*/*M* ≈ 1300, according to the 10% valley definition) was insufficient for complete separation of all mass 26 and 27 ionic species. As such, we consider the results obtained by ToF‐SIMS as semiquantitative, in contrast to the values obtained by NanoSIMS. However, ToF‐SIMS is superior to NanoSIMS in the achievable size of the field of view that can be scanned without significant lens aberration (up to 500 × 500 μm^2^ vs *c*. 100 × 100 μm^2^, respectively). Accordingly, we utilized ToF‐SIMS for obtaining survey images with high throughput and NanoSIMS for high‐accuracy isotope analysis within particular measurement areas predefined by ToF‐SIMS.

#### NanoSIMS

NanoSIMS measurements were performed on an NS50L instrument (Cameca, Gennevilliers, France) at the Large‐Instrument Facility for Environmental and Isotope Mass Spectrometry of the University of Vienna. Before data acquisition, analysis areas were presputtered utilizing a high‐intensity, slightly defocused Cs^+^ ion beam (100–200 pA beam current, *c*. 1.5 μm spot size) to a primary ion fluence of 6.2E16 ions per cm^2^, which enabled the establishment of the steady‐state secondary ion signal intensity regime. Data were acquired as multilayer image stacks by sequential scanning of a finely focused Cs^+^ primary ion beam (*c*. 80 nm probe size at 2 pA beam current) over areas between 60 × 60 and 73 × 73 μm^2^ at 512 × 512 pixel image resolution and a primary ion beam dwell‐time of 7.5 ms (pixel × cycle)^−1^. The detectors were positioned to enable parallel detection of ^12^C^−^, ^12^C_2_^−^, ^12^C^14^N^−^, ^12^C^15^N^−^, ^31^P^−^ and ^32^S^−^ secondary ions. The mass spectrometer was tuned to achieve a mass resolving power of > 9000, according to Cameca's definition (Hoppe *et al*., 2013), which enabled selective detection of the targeted secondary ion species (i.e. ^12^C^14^N^−^ and ^12^C^15^N^−^) without contributions from any other potentially formed secondary ions species at M26 and M27 (Table S2).

NanoSIMS images were processed using the WinImage software package v.2.0.8 provided by Cameca. Before stack accumulation, the individual images were aligned to compensate for positional variations arising from primary ion beam and/or sample stage drift. Secondary ion signal intensities were dead time corrected on a per‐pixel basis. Nitrogen isotope composition images displaying the ^15^N/(^14^N + ^15^N) isotope fraction, designated as ^15^N atom%, were inferred from the ^12^CN^−^ secondary ion signal intensity distribution images *via* per‐pixel calculation of ^12^C^15^N^−^/(^12^C^14^N^−^ + ^12^C^15^N^−^) intensity ratios. Natural isotopic abundance values were obtained by measurement of unlabelled control samples. Regions of interest (ROIs) were manually defined utilizing (^12^C^14^N^−^ + ^12^C^15^N^−^)/^12^C_2_^−^ signal intensity ratio maps as an indicator of the relative nitrogen‐to‐carbon elemental ratio, which visualizes characteristic tissue and intracellular structures.

### Fungus culture

Fungus cultures were obtained from the tissue as well as from ant waste of six living pseudobulbs inhabited by *Azteca* cf *velox*. Fungal isolation from ant waste was done as described in Voglmayr *et al*. (2011) and Nepel *et al*. (2014). For isolation of fungi from living host tissue of pseudobulbs, a slightly modified protocol of Bougoure *et al*. (2005) was used. Thin sections of surface sterilized pseudobulbs were placed on 2% malt extract agar (MEA) plates supplemented with antibiotics (0.1% Streptomycin, 0.1% Penicillin G; Sigma Aldrich). Hyphal tips from outgrowing hyphae were transferred to new MEA plates. Once free from bacterial and yeast contamination, hyphae were transferred onto new plates and grown at room temperature.

### DNA extraction and sequencing

Mycelium growth and DNA extraction was performed according to Voglmayr & Jaklitsch (2011). The nuclear ITS‐partial LSU rDNA was amplified and sequenced as a single stretch as described in Voglmayr *et al*. (2011) from 21 pure fungus cultures. Taxonomic identification of the sequences was done using a Blast search in GenBank. All sequences are deposited in GenBank (http://www.ncbi.nlm.nih.gov/genbank/). Strains and GenBank accession numbers are listed in Table 1.

**Table 1:** Fungal pure cultures of six *Caularthron bilamellatum* pseudobulbs inhabited by *Azteca* cf *velox*.

Source	Taxon (% ITS Blast similarity)	Subclass	Order	Isolates	ITS‐LSU GenBank accession
T	*Longitudinalis nabanheensis* (95.3%)	Sordariomycetidae	Glomerellales	1	MZ545396
T	*Fusarium* sp. MX271 (99.8–100%)	Sordariomycetidae	Hypocreales	3	MZ545399, MZ545400, MZ545401
T	Hypocreales sp. MS556 (100%)	Sordariomycetidae	Hypocreales	1	MZ545397
T	*Ijuhya vitellina* (85.6%)	Sordariomycetidae	Hypocreales	1	MZ545398
T	*Purpureocillium lavendulum* (100%)	Sordariomycetidae	Hypocreales	2	MZ545387, MZ545389
T	*Purpureocillium lilacinum* (100%)	Sordariomycetidae	Hypocreales	1	MZ545388
T	*Xenoacremonium falcatum* (100%)	Sordariomycetidae	Hypocreales	2	MZ545390, MZ545391
T	*Acrodontium griseum* (99.8%)	Sordariomycetidae	*Incertae sedis*	2	MZ545394, MZ545395
T	*Sporothrix* sp. TMS‐2011 (90.7%)	Sordariomycetidae	Ophiostomatales	2	MZ545392, MZ545393
S	Chaetothyriales sp. MACpB (98.9%)	Chaetothyriomycetidae	Chaetothyriales	1	MZ545403
S	*Cladophialophora scillae* (91%)	Chaetothyriomycetidae	Chaetothyriales	1	MZ545407
S	*Cladophialophora* sp. KO‐groupL 2014 (91%)	Chaetothyriomycetidae	Chaetothyriales	1	MZ545402
S	*Cladosporium* spp. (100%)	Dothideomycetidae	Cladosporiales	2	MZ545405, MZ545406
S	*Teratosphaeria* sp. F1920 (96%)	Dothideomycetidae	Mycosphaerellales	1	MZ545404

Samples were taken from living tissue (T) and the inner surface of hollow pseudobulbs (S) and cultured on 2% MEA agar plates. Taxonomic affiliation was determined by sequencing the complete ITS1‐5.8S‐ITS2 nuclear ribosomal DNA and subsequent NCBI Blast search.

### Statistics

Statistical analysis was performed with RStudio team (2020) v.2022.07.2. The overall effect of treatment and time points was tested with a generalized linear model (GLM). Pairwise comparisons were made with a Kruskal–Wallis rank‐sum test and a subsequent Dunn's *post hoc* analysis.

## Results

### Pseudobulb morphology

**Fig. 2:**
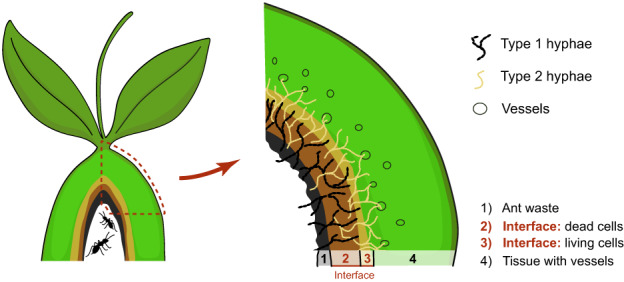
Schema of a pseudobulb of *Caularthron bilamellatum* colonized by ants. Hyphae of type 1 (Capnodiales, Chaetothyriales and Mycosphaerellales) were found in ant waste and dead cells originating from the former water storage tissue, only rarely in living tissue. Hyphae of type 2 (Hypocreales) mainly colonized the living tissue but were also found in the dead cell layers and rarely in ant waste. The area where both types of hyphae occur together is called ‘interface’.

The most prominent anatomical feature of the cavity surface is the large thick‐walled and suberized dead cells of the desiccated former water storage tissue (Gegenbauer *et al*., 2012 – fig. 2C). They sometimes exceed 1 mm in diameter and are visible to the naked eye as a rough surface, especially in the apical region. These cells form deep depressions in which ant waste accumulates (Fig. 1c). Closed pseudobulbs that had not formed a slit miss those structures on the cavity surface. Instead, it was partially covered with the residues of the tissue that had desiccated (Fig. S2a).

### Ant inhabitants and appearance of the pseudobulb interior

Of the six sampling plots, two were inhabited by *Azteca* cf *velox* (Dolichoderinae), which was the most common ant species encountered. Other ant species found in one plot each were another larger *Azteca* sp., *Camponotus atriceps* (Formicinae) and *Pheidole* sp. (Myrmicinae). One plot turned out to be ant free but with signs of former ant presence in pseudobulbs, suggesting recent abandonment.

In each plot, 10–15 pseudobulbs were examined. The appearance of the interior of the pseudobulbs varied depending on the inhabiting ant species (Table S1). The abandoned ant‐free pseudobulbs (Fig. S2b) had the same characteristics as the *Azteca*‐inhabited ones. The ones inhabited by *Camponotus* exhibited a thick, dark brown and mud‐like layer of ant waste comprised of dead ants, insect remains and fungal hyphae and were populated by a variety of organisms like coccids, small snails, nematodes and protists (Fig. S2c). The inside of all pseudobulbs inhabited by *Pheidole* was of light colour, exhibiting a rough surface but with little to no detritus (Fig. S2d). In the pseudobulbs of the three plots inhabited by different *Azteca* species, the waste area in the apical region was blackish‐brown and heavily pervaded by melanized fungal hyphae (Fig. S2e–g). Cardboard‐like structures (=carton) which are typical for nests of *Azteca* ants were present.

### Identity of fungi and distribution

At least two strikingly distinct hyphal types were commonly encountered in ant‐inhabited pseudobulbs.

Type 1 comprises thick and distinctly septate melanized hyphae with a diameter of 4–8 μm. These hyphae pervaded the ant waste on the surface of the pseudobulb cavity and the layers of dead cells. They also grew through numerous pits 1–3 cell layers into the adjacent living tissue (Fig. 1e,h). We identified the isolates as members of the Ascomycota from Chaetothyriales, Cladosporiales and Mycosphaerellales orders (Table 1; Fig. S3d). Obviously, these melanized hyphae were responsible for the characteristic dark brown colour of pseudobulb cavities inhabited by ants of the genus *Azteca* (Figs 1b, S2e–g), and *Camponotus atriceps* (Fig. S2c). Very few to no such hyphae were observed in pseudobulbs inhabited by *Pheidole* sp., which explains the light colour of the chamber surface (Table S1; Fig. S2d).

Type 2 are thin, hyaline and indistinctly septate hyphae with a diameter of 1–2 μm. Most isolates from this type could be assigned to the ascomycete order Hypocreales (Fig. 1d,f,g; Table 1). Hyphae of this type were observed to grow along the cell walls (Figs 1d, S3a–c), several layers deep into the living cells and into cells of the vascular bundles (Fig. 1g). Few hyphae of this type were also found in the dead cells together with thick melanized hyphae of type 1 and from time to time in the detritus covering the pseudobulb cavity (Fig. 1h). All visible endophytes were, without exception, limited to the cell layers of the innermost third of the pseudobulb tissue and never found in the part close to the epidermis. They were especially prominent in *Azteca* and *Pheidole* sp. inhabited ones, while being less frequent in abandoned pseudobulbs and those colonized by *Camponotus atriceps* (Table S1).

Both hyphae types co‐occurred in the layers of dead cells and 1–3 layers in the living tissue. This zone is called the ‘interface zone’ (Fig. 2).

Closed pseudobulbs, which had failed to form a natural opening, were completely free of fungal hyphae on the chamber surface and in living tissue.

### IRMS of pseudobulbs and leaves

**Fig. 3:**
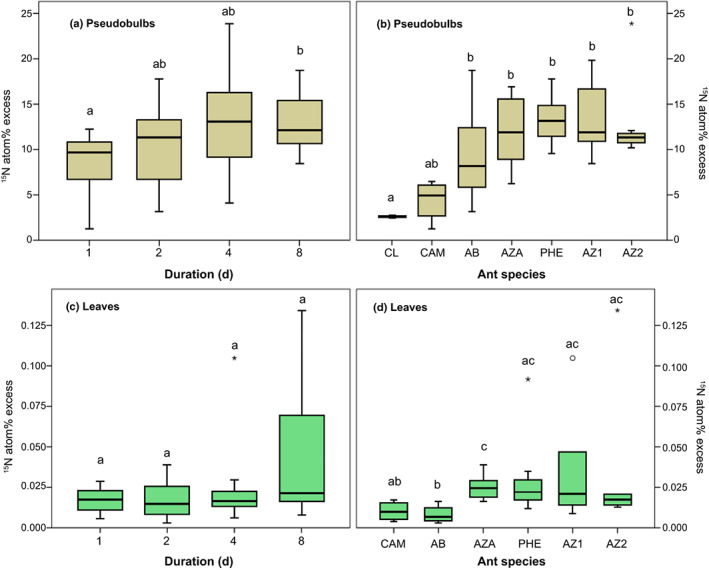
^15^N Isotope ratio mass spectrometry (IRMS) data for *Caularthron bilamellatum* pseudobulbs (pbs) and leaves. Box plots show the median as horizontal line, interquartile ranges as coloured box and whiskers as minimum and maximum values. Rings denote outliers and asterisks extreme outliers. Different letters indicate significant differences between groups (Kruskal‐Wallis rank‐sum test with a Dunn *post hoc* analysis). (a) Time series for labelled pbs from 1 to 8 d. Pbs from all plots pooled. The incubation time had a significant effect on ^15^N APE in pseudobulbs (pbs: GLM estimate 1.53, *t*‐value 2.1, df = 40, *P* = 0.043, whereas day 1 and 8 differed significantly *P* = 0.023, Kruskal–Wallis rank‐sum test with a Dunn *post hoc* analysis). (b) Comparison of pbs inhabited by different ant species, each pooled across time series 1–8 d. CL, closed sterile (*n* = 2); CAM, *Camponotus atriceps* (*n* = 4); AB, uninhabited but with signs of former ant presence (*n* = 7); AZA, *Azteca* sp. (*n* = 8); PHE, *Pheidole* sp.; AZ1 (*n* = 7) and AZ2 (*n* = 7), *Azteca* cf *velox*. Significant differences were found between CL and CAM vs PHE, AZ1, AZ2, AZA (higher ^15^N APE, *P* < 0.01, df = 6; Kruskal–Wallis rank‐sum test with a Dunn *post hoc* analysis). (c) Time series for leaves (all *n* = 6 except for AZA with *n* = 8) carried by pseudobulbs shown in (a). (d) Comparison of leaves carried by pbs shown in (b). No leaf samples for closed pbs could be obtained. AB had a significantly lower ^15^N APE than PHE and AZ1, AZ2, AZA (*P* < 0.01, df = 6; Kruskal–Wallis rank‐sum test with a Dunn *post hoc* analysis).

^15^N enrichment of ant‐inhabited pseudobulbs after labelling was highly significant compared with the control (atom% ^15^N pseudobulbs: GLM estimate 11.04, *t*‐value 0.22, df = 48, *P* < 0.001) at each time point (atom% ^15^N pseudobulbs day 1, 2, 4, 8: GLM estimate 2.91, *t*‐value 5.69, df = 48, *P* < 0.001). Already 24 h after label application, a considerable ^15^N uptake could be measured (atom% excess – ^15^N APE 8.5 ± 1.3 SE; Table S3). The incubation time had an effect on ^15^N enrichment of the pseudobulb tissue (^15^N APE: GLM estimate 1.53, *t*‐value 2.1, df = 40, *P* = 0.043) with a significantly higher enrichment at day 8 compared with day 1 (*P* = 0.023, Kruskal–Wallis rank‐sum test with a Dunn *post hoc* analysis; Fig. 3a; Table S3). Closed pseudobulbs without ant‐contact (CL) showed significantly lower ^15^N enrichment than open ones accessible by ants (GLM estimate 8.88, *t*‐value 2.6, df = 40, *P* = 0.014; Fig. S4). Closed pseudobulbs (CL) and those inhabited by *Camponotus atriceps* (CAM) had a significantly lower ^15^N uptake (in APE) than pseudobulbs inhabited by *Pheidole* sp. (PHE), *Azteca* cf *velox* (AZ1 and AZ2; *P* < 0.01, df = 6; Kruskal–Wallis rank‐sum test with a Dunn *post hoc* analysis; Fig. 3b).

The translocation of ^15^N into leaves was quick and ^15^N enrichment significant compared with unlabelled plant tissue at all time points (atom% ^15^N leaves day 1, 2, 4, 8: GLM estimate 0.009, *t*‐value 3.61, df = 43, *P* < 0.001). However, labelling duration had no significant impact on ^15^N enrichment (Fig. 3c). Interestingly, ^15^N enrichment of leaves of the abandoned pseudobulbs (AB) did not differ significantly from *Camponotus atriceps* (CAM) inhabited ones. Both had a significantly lower ^15^N APE than pseudobulbs inhabited by *Pheidole* sp. (PHE) and all *Azteca* species (AZ1, AZ2, AZA; *P* < 0.01, df = 6; Kruskal–Wallis rank‐sum test, Dunn *post hoc* test; Fig. 3d; Table 2). Unfortunately, no data on leaf samples of the two closed pseudobulbs could be obtained.

**Table 2:** Mean ^15^N atom% excess (APE) ±SE in labelled pseudobulbs and corresponding leaves of *Caularthron bilamellatum* for each sampling plot separately.

Ant species plot designation	None/closed (CL)	*Camponotus atriceps* (CAM)	Abandoned (AB)	*Azteca* sp. (AZA)	*Pheidole* sp. (PHE)	*Azteca* cf *velox* (AZ1)	*Azteca* cf *velox* (AZ2)
Pseudobulbs Sample size	2.6 ± 0.15 *n* = 2	4.4 ± 1.2 *n* = 4	9.5 ± 2.1 *n* = 7	12.0 ± 1.4 *n* = 8	13.3 ± 1.1 *n* = 7	13.6 ± 1.6 *n* = 7	13.0 ± 1.8 *n* = 7
Leaves Sample size	–	0.01 ± 0.003 *n* = 6	0.008 ± 0.002 *n* = 6	0.025 ± 0.003 *n* = 7	0.031 ± 0.010 *n* = 6	0.036 ± 0.015 *n* = 6	0.036 ± 0.020 *n* = 6

Closed pbs failed to form an opening for ants and had never been colonized by ants. The plot with abandoned pbs was free of ants at the sampling time, but with clear signs of previous ant colonization. ^15^N APE from closed pbs and the ones inhabited with *C. atriceps* differed significantly from pbs colonized by *Pheidole* sp. and the *Azteca* species (AZ1, AZ2, AZA), (*P* = 0.01, df = 6, Kruskal–Wallis rank‐sum test with a Dunn *post hoc* analysis). ^15^N APE of leaves from the abandoned pseudobulbs were significantly different from leaves of the pbs colonized with *Pheidole* sp. and *Azteca* species (*P* = 0.007, df = 5, Kruskal–Wallis rank‐sum test with a Dunn *post hoc* analysis). No leaf samples were obtained from the closed group.

### ToF‐SIMS

**Fig. 4:**
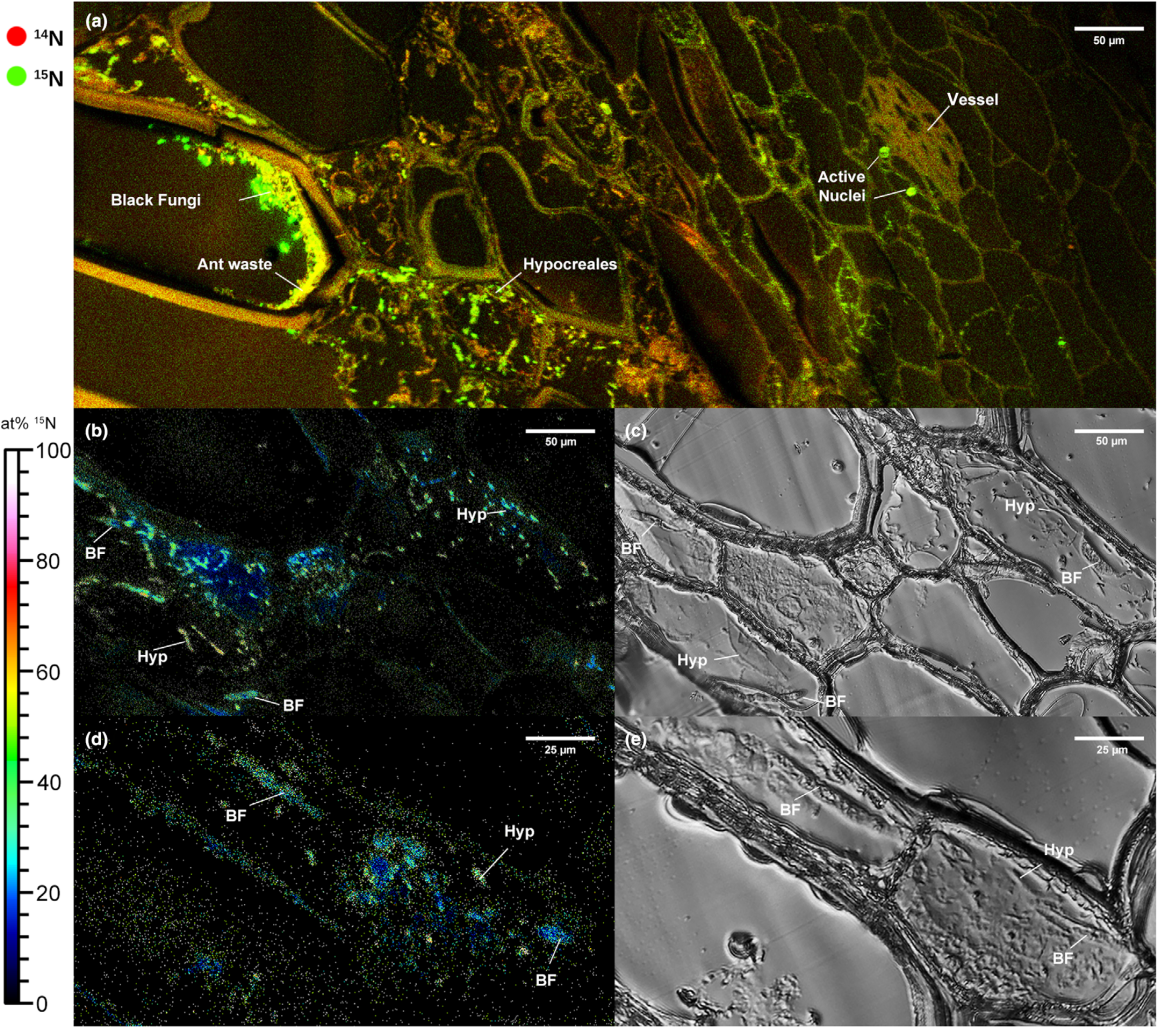
Secondary ion mass spectrometry images of ^15^N uptake in *Caularthron bilamellatum* inhabited by *Azteca* cf *velox*. The false‐colour scale displays at% ^15^N values ranging from 0 (black) to 100 (white). (a) Qualitative ToF‐SIMS image of pseudobulb (pb) tissue, cross section. Giant cells bordering the pb chamber can be seen on the left side, and the epidermis would be towards the right (not visible). Red, ^12^C^14^N^−^ (natural abundance); green, ^12^C^15^N^−^ (label). Pbs were labelled by placing agar discs containing a high molecular weight ^15^N source on the surface of the hollow chamber for 24 h. Thick hyphae (black fungi) in the detritus layer and in dead giant cells and thinner hyphae (Hypocreales) in the innermost living cell layer exhibit strong ^15^N uptake. ^15^N is transported towards active cell nuclei and into a vessel. Note that signal intensities are biased by mass interferences. (b, d) Semiquantitative ToF‐SIMS image of a longitudinal section through the innermost living cells rich in endophytes displaying ^12^C^15^N^−^ normalized to the sum of ^12^C^14^N^−^ + ^12^C^15^N^−^ after a labelling period of 8 d. Unlabelled background (natural abundance) is shown in black, moderate enrichment up to 20 atom% ^15^N in blue. Fungal hyphae are displayed as turquoise to yellow dots and exhibit massive enrichment of 50–60 atom% ^15^N. (c, e) Light microscopy image of the corresponding regions. Thick hyphae (black fungi) and thin hyphae (Hypocreales) can be seen within the same cell. All strong ^15^N signals can be correlated to hyphae‐like structures but not all hyphae incorporated ^15^N. BF, black fungi; Hyp, Hypocreales.

A large‐area composite ToF‐SIMS image (900 × 300 μm^2^) of a *C. bilamellatum* pseudobulb inhabited by *Azteca* cf *velox* and labelled with ^15^N for 24 h gives an overview of the label distribution. It shows a highly heterogeneous ^15^N uptake with pronounced hotspots visible in the detritus layer and in dead cells. Cross sections of thick hyphae (type 1) and smaller spots probably depicting the small hyaline Hypocreales (type 2) could be resolved (Fig. 4a). However, many structures identified as hyphae remained unlabelled. Apart from hyphae, ^15^N was translocated deep into living tissue along cell walls and the active nuclei close to a vessel about eight layers of living cells inwards from the pseudobulb chamber (Fig. 4a). IRMS measurement of the corresponding bulk samples yielded 10.3 APE ^15^N for pseudobulb tissue and 0.009 APE ^15^N for leaves.

From a second sample inhabited by *Azteca* cf *velox* which had been labelled for 8 d, an area of 500 × 500 μm^2^ was imaged. ^15^N was almost exclusively found in hyphae, which could clearly be attributed to either thick type 1 or thin type 2 (Hypocreales). The corresponding morphological structures were validated in LM images (Figs 4b–e, S5) The ^15^N label content in thin hyphae reached values of up to nominally 50 atom% but varied largely. The IRMS determined bulk values for this sample were 8.4 APE ^15^N for pseudobulb tissue and 0.05 APE ^15^N for leaves.

### NanoSIMS

**Fig. 5:**
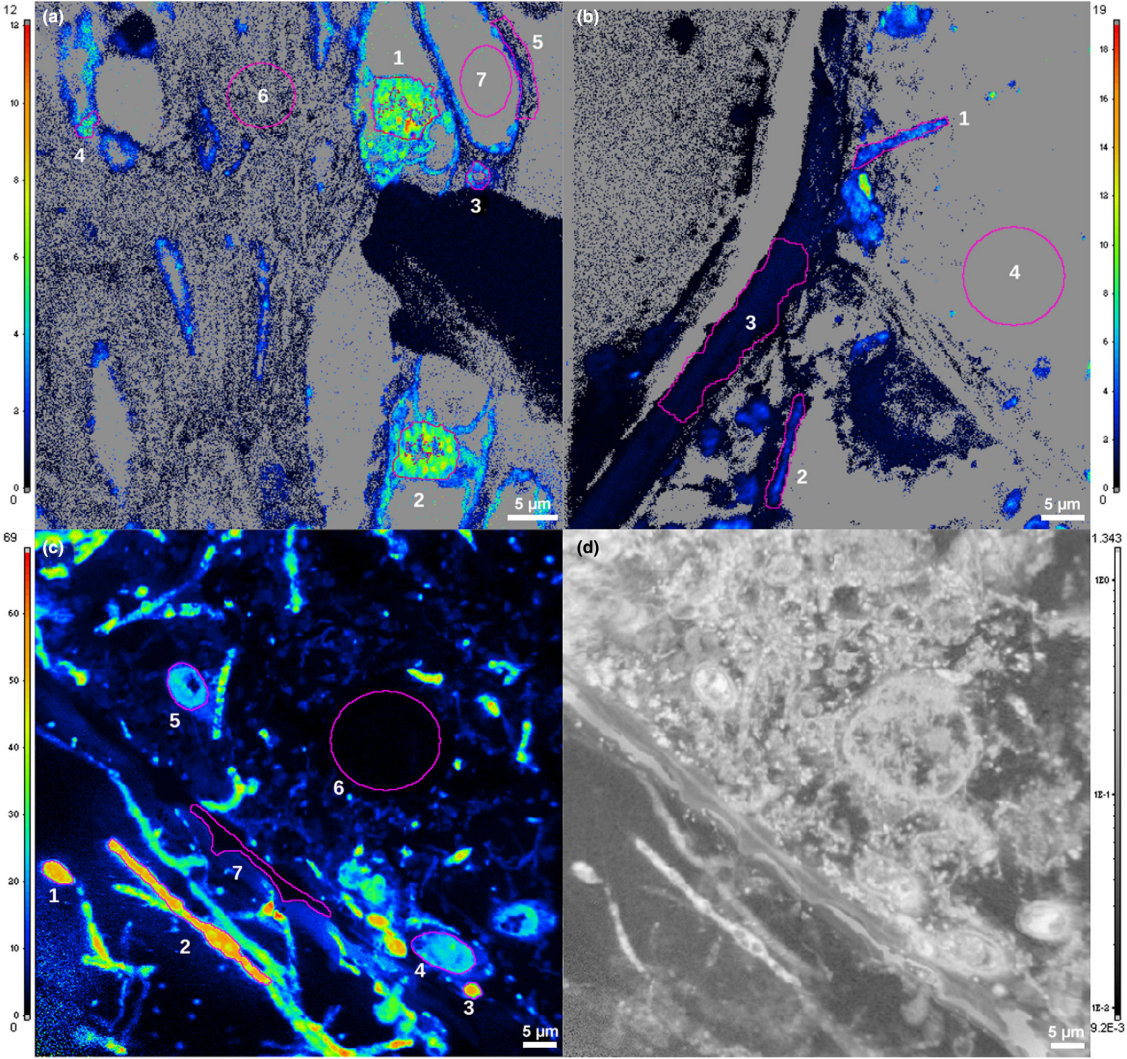
Quantitative NanoSIMS isotope imaging of *Caularthron bilamellatum* pseudobulb inhabited by *Azteca* cf *velox* and labelled with ^15^N either for 24 h (a, b) or 8 d (c). The false‐colour scale displays atom% ^15^N values, regions of interest (ROIs) are marked in purple, and the corresponding δ^15^N and atom% excess (APE) values are given in Table 3. (a) Region around a vessel about six cell layers distant from the hollow chamber. ^15^N is strongly incorporated into active nuclei reaching 5 APE (a1,2), into hyphae with 2.9 APE (a3) and in the protoplast of a vessel with up to 3.5 APE (a4). Cell walls displayed much lower uptake of 0.5 APE (a5) or remained almost unlabelled at 0.02 APE (a6), as did the protoplast of a cell outside the vessel with 0.15 APE (a7). (b) Innermost living cell bordering the pseudobulb chamber in close vicinity to where the labelling substance was placed. ^15^N is incorporated in hyphae (oval and bar‐like structures) to 2–3 APE (b1,2). Thick cell walls show a slight enrichment of 0.45 APE (b3) while the protoplast remained nearly unlabelled at 0.06 APE (b4). (c) Interface harbouring thick and thin hyphae, 2–3 cell layers distant from the pseudobulb cavity. Thin Hypocreales hyphae (c1–3) incorporated up to 50 APE, thick hyphae of black fungi (c4,5) up to 20 APE. An inactive cell nucleus, 0.71 APE (c6) and the cell wall, 2.3 APE (c7) incorporated far lower amounts of ^15^N. (d) (^12^C^14^N^−^ + ^12^C^15^N^−^)/^12^C_2_^−^ secondary ion intensity ratio image corresponding to the area displayed in (c), revealing intracellular structures and hyphae with high nitrogen content. Grey areas in (a, b) refer to pixels where an unbiased atom% ^15^N visualization is not feasible due to inferior counting statistics, emerging from weak ^12^CN‐ secondary ion signal intensities (mainly within resin areas).

The same two samples were used for close‐up views on the cellular level in NanoSIMS. Of particular interest were the vessel deep inside the pseudobulb tissue, the innermost living cell layer bordering the pseudobulb cavity and the interface zone where both hyphae types co‐occurred. After a labelling time of 24 h, vessel protoplast exhibited a considerable enrichment (0.15–3.5 APE ^15^N) while cell walls only showed moderate‐to‐low values (0.02–0.5 APE ^15^N). A ring‐like structure representing a cross‐sectioned thin fungal hyphae (type 2) was also highly enriched (2.9 APE ^15^N). The highest ^15^N enrichment was observed in the two active cell nuclei near the vessel (5.0 APE ^15^N and 4.8 APE ^15^N; Fig. 5a; Table 3a).

**Table 3:** NanoSIMS determined atom% excess (APE) and δ^15^N (‰) values of *Caularthron bilamellatum* pseudobulb (pb) inhabited with *Azteca* cf *velox* within the particular regions of interest (ROI) shown in Fig. 5.

ROI (a)	No. 1	No. 2	No. 3	No. 4	No. 5	No. 6	No. 7
Area	Cell nucleus	Cell nucleus	Thin hyphae	Vessel protoplast	Cell wall	Vessel cell wall	Protoplast
^15^N APE (%)	5.0	4.8	2.9	3.5	0.5	0.02	0.15
δ^15^N (‰)	14 403	13 877	8317	9898	1393	52	414

ROI (b)	No. 1	No. 2	No. 3	No. 4
Area	Thin hyphae	Thin hyphae	Cell wall	Protoplast
^15^ N APE (%)	3.0	1.4	0.42	0.06
δ^15^N (‰)	8353	3783	1162	175

ROI (c)	No. 1	No. 2	No. 3	No. 4	No. 5	No. 6	No. 7
Area	Thin hyphae	Thin hyphae	Thin hyphae	Thick hyphae	Thick hyphae	Cell nucleus	Cell wall
^15^N APE (%)	48.0	49.0	45.5	20.3	17.2	0.71	2.3
δ^15^N (‰)	253 847	264 249	229 438	69 637	56 755	1964	6491

Pbs were labelled by placing an agar disc with an organic ^15^N source into the apex of hollow pbs for 24 h (a, b) or 8 d (c). (a) Vessel about six cell layers inwards from the pb cavity. (b) Innermost living cell layer bordering the pbs cavity. (c) Living cells rich in endophytic fungi 2–3 layers from the pb cavity.

Within the innermost living cell layer close to the pseudobulb cavity, hyphae of type 2 (Hypocreales) were strongly labelled (3.0, 1.4 APE ^15^N, respectively). Cell walls had an enrichment of 0.4 APE ^15^N. An area free of plasmatic structures and interpreted as the vacuole, exhibited the lowest values (0.06 APE ^15^N; Fig. 5b; Table 3b).

In the interface 2–3 layers from the pseudobulb cavity, we found a very high label accumulation in both hyphae types after 8 d of label incubation. Remarkably, the thin hyphae (type 2, Hypocreales) reached much higher values (49, 48 and 45.5 APE ^15^N) than the thick hyphae (type 1; 20.3, 17.2 APE ^15^N). The cell nucleus (0.71 APE ^15^N) and cell wall (2.3 APE ^15^N) exhibited a significantly lower, but still substantial enrichment (Fig. 5c; Table 3c). Label accumulations could be correlated with intracellular structures visible in the (^12^C^14^N^−^ + ^12^C^15^N^−^)/^12^C_2_^−^ signal intensity ratio images (Fig. 5d).

## Discussion

By combining bulk IRMS data and visualization of ^15^N uptake by means of ToF‐SIMS and NanoSIMS, we could demonstrate that the agar‐bound label placed on ant detritus in hollow pseudobulbs of *C. bilamellatum* was mainly incorporated into specific structures and did not diffuse uniformly between cells. This rules out passive diffusion as the main path of nitrogen transfer. Instead, apoplastic transport along cell walls and enrichment of fungal hyphae was observed, which indicates that fungal hyphae are particularly absorptive structures to take up ^15^N from the ant waste site. ^15^N uptake was considerable and a part of it was even transferred into the leaves within only 24 h.

Single hyphae sampled from the inner surface of hollow pseudobulbs where the ant waste was deposited and from the pseudobulb tissue could be cultivated and were assigned to respective fungal orders. The thin hyaline endophytes (type 2) were identified as members of Hypocreales, an order with different lifestyles ranging from plant and insect pathogens to symbiotic endophytes (Saikkonen *et al*., 1998; Wu & Cox, 2021). The cultures grown from septate and melanized single hyphae (type 1) were assigned to Capnodiales, Chaetothyriales and Mycosphaerellales, orders which belong to the so‐called ‘black fungi’. Members of this group of phylogenetically unrelated fungi have in common the presence of melanin in the cell wall, the formation of yeast‐like daughter cells under certain environmental conditions, and their ability to withstand hostile environments (de Hoog & Hermanides‐Nijhof, 1977; Gostinčar *et al*., 2012). This ability makes black fungi frequent associates of ants with a more or less mutualistic relationship (Schlick‐Steiner *et al*., 2008; Voglmayr *et al*., 2011; Mayer *et al*., 2018; Moreno *et al*., 2019; Blatrix *et al*., 2021).

First evidence for an involvement of an ant‐associated fungus in nitrogen transfer from ant made carton outside the stem to the plant tissue provided a study on the ground‐rooting ant‐plant *Hirtella physophora* (Chrysobalanaceae; Leroy *et al*., 2017). The authors classified the fungus as belonging to Chaetothyriales and suggested that the hyphae outside and the ones inside the plant tissue belong to the same species. The occurrence of > 1 fungal strain involved in nutrient uptake distinguishes our study considerably from the findings in myrmecophytic *Hirtella physophora*. In that ant‐plant, inhabiting ants form galleries stabilized by a fungus classified as member of Chaetothyriales, which mediated transfer of nitrogen from carton to the plant (Leroy *et al*., 2011, 2017). The authors found endophytic hyphae inside the plant cells and propose that the hyphae on the gallery outside the plant tissue and the ones in the plant tissue belong to the same fungal species (Leroy *et al*., 2017).

In *C. bilamellatum*, however, a high abundance of black fungi in the ant waste and the adjacent layer of dead cells originating from the now dried‐up original water storage tissue was not correlated with either nutrient uptake or translocation into leaves. Instead, the best predictor for the average nutrient uptake was the abundance of hyphae assigned to Hypocreales forming a net of hyphae in the living tissue but grew also – though less abundant – in the dead cell layer and rarely also directly into the ant waste. High abundance of Hypocreales and low abundance of black fungi resulted in a higher uptake rate than in the reverse case with high abundance of black fungi and low abundance of Hypocreales. In the interface zone in which both hyphal types co‐occur, Hypocreales exhibited an ^15^N enrichment more than twice as high as hyphae of black fungi. However, as the incorporation of ^15^N is only expected in newly grown hyphal cells, the difference in enrichment probably reflects the very slow growth rate in black fungi compared with Hypocreales (Abdollahzadeh *et al*., 2020; Quan *et al*., 2020).

From that pattern, we deduce that in *C. bilamellatum* Hypocreales are especially important for rapid nutrient transport into living tissue and vascular bundles, and from there into leaves and reproductive parts due to their faster growth and endophytic lifestyle. Remarkably, though variation among replicates was substantial, we found the highest label translocation rates to leaves when both, black fungi and Hypocreales, had a high abundance in the interface zone. This area seems to be an important interface at a fungus–fungus interaction level with a synergistic effect for nutrient transfer. To explain the peaks, a scenario with two simultaneous uptake pathways is conceivable: One pathway involves black fungi (type 1) taking up the high molecular weight ^15^N label from the site of the ant waste and processing it in their intermediary metabolism. Amino acids are then released back into the apoplast of cells of interface zone where both hyphae types co‐occur. From there, endophytic Hypocreales could absorb the ^15^N labelled amino acids and transfer them to the vascular bundles. The second pathway is direct *via* endophytic Hypocreales hyphae that have been growing into the ant waste. As Hypocreales are less abundant in the layer of dead cells, and even less in the waste than in living cells, direct nutrient uptake will result in a lower amount of ^15^N transfer than a 2‐way transfer involving both hyphal species at the same time. Due to their rapid growth and hyphal network in the living pseudobulb tissue, Hypocreales are most likely responsible for the amazing speed of nutrient uptake found in our previous study (Gegenbauer *et al*., 2012).

Endophytically growing Hypocreales are frequently encountered in plant roots. Recently, for several root endophytes with entomopathogenic lifestyle (e.g. *Metarhizium* spp. and *Beauveria bassiana*, both Hypocreales, Clavicipitaceae), nitrogen transfer from dead insects in the soil to plants *via* a fungal mycelium has been demonstrated (Behie *et al*., 2012; Behie & Bidochka, 2014). In exchange for delivering nitrogen, *Metarhizium* receives photosynthetically fixed carbon (Behie *et al*., 2017; Barelli *et al*., 2019). Thus, nonmycorrhizal Hypocreales colonizing plant roots can perform mycorrhiza‐like reciprocal nutrient exchange.

In general, hypocrealean endophytes are not strictly bound to root symbiosis but can also use other niches, sometimes even simultaneously (Leger & Wang, 2020); therefore, the mechanism underlying endophyte‐mediated nitrogen transfer in the pseudobulb tissue of *C. bilamellatum* is expected to be comparable to that in roots. Ant waste in the hollow pseudobulbs acts as an equivalent to soil in terms of nutrient source for this epiphytic orchid with no contact with the ground, and the stem (=pseudobulb)‐born endophytic network of Hypocreales mediating nutrient transfer from ant‐derived waste to the host plant tissue may be functionally equivalent to mycorrhizal mycelia in roots.

Unfortunately, to date, we do not know whether carbon from plant photosynthates is reciprocally transferred into the hyphae. Nor do we know which of the six Hypocreales strains cultivated from the pseudobulb mediates nutrient transfer. Candidates are *Purpureocillium lilacinum* and *P. lavendulum* (Hypocreales, Ophiocordycipitaceae) isolated from the tissue of several *C. bilamellatum* pseudobulbs, which are known to enhance plant growth and reproductive tissue development in cotton plants as root endophytes (Castillo Lopez & Sword, 2015). Fungal endophytes may improve host resilience to abiotic stress like drought and heat (Rodriguez *et al*., 2008; González‐Teuber *et al*., 2018; Moghaddam Hosseyni *et al*., 2021), both of which are – together with nutrient deficiency – extreme in the natural environment of *C. bilamellatum*.

Information on the variation and direction of nutrient flux within the plant throughout the annual growth cycle is lacking so far but the mature pseudobulbs examined may not exclusively be a nitrogen sink as we demonstrated in this study. They may also act as a nutrient source for adjacent immature pseudobulbs, depending on nutrient distribution and demand within the entire plant (Zotz, 1999). In this scenario, nitrogen would rather be translocated to stronger nutrient sinks within the plant like the emerging new growth than stored in mature pseudobulbs and their leaves. This might be a reason for the strong variation in ^15^N transfer to leaves as well as the lack of ^15^N accumulation over time above a certain baseline.

### Conclusion

We were able to demonstrate that two types of fungi – exophytic and endophytic ones – mediate the remarkably rapid transfer of nutrients from ant waste to the plant tissue in the epiphytic orchid *C. bilamellatum*. This is the first evidence that a network of two different fungi results in a synergism in terms of nutrient uptake. While studies on nutrient transfer *via* mycorrhiza in roots are numerous, much less is known about the role of endophytic fungi in the stem of plants for nutrient provision. In nutrient‐deficient habitats like a tropical rainforest canopy, the capacity to use nutrients from insect waste may be crucial for survival and reproduction for plants with an arboreal lifestyle.

## Competing interests

None declared.

## Author contributions

CG, VEM, AR and GZ designed the experiment; CG performed the field work and stable isotope labelling experiment in Panama; CG and AB did the anatomical sectioning and light microscopy; VEM created SEM and CT images; CG, VEM and HV performed the fungal pure culture isolation; HV performed DNA extraction, sequencing and sequence data analysis of fungi; CG and AR analysed the IRMS data; MCS participated in the preparation of the samples for SIMS analyses; AS performed the NanoSIMS analysis with contribution from CG; MK did ToF‐SIMS analysis with contribution from CG; CG and VEM wrote the manuscript with contributions from all authors.

## Supporting information


**Fig. S1** Annual growth cycle and hypothetical nutrient transfer.
**Fig. S2** Morphology of pseudobulb chamber inhabited by different ant species.
**Fig. S3** SEM images of pseudobulbs inhabited by *Azteca* cf *velox*.
**Fig. S4** ^15^N enrichment in open and closed pseudobulbs.
**Fig. S5** Semiquantitative ToF‐SIMS image of a longitudinal section through the innermost living cells rich in endophytes displaying ^12^C^15^N^−^ normalized to the sum of ^12^C^15^N^−^ + ^12^C^14^N^−^ after a labelling period of 8 d.
**Table S1** Sampling plots of *Caularthron bilamellatum* pseudobulbs and associated ant species.
**Table S2** *M*/Δ*M* values of negatively charged secondary ion species.
**Table S3** Labelling time and enrichment of pseudobulbs and leaves.
Please note: Wiley is not responsible for the content or functionality of any Supporting Information supplied by the authors. Any queries (other than missing material) should be directed to the *New Phytologist* Central Office.

## Data Availability

All obtained sequences were deposited in GenBank (accession nos. MZ545387–MZ545407). Additional data that support the findings of this study are available in the Supporting Information of this article.

## References

[nph18761-bib-0001] Abdollahzadeh J , Groenewald JZ , Coetzee MPA , Wingfield MJ , Crous PW . 2020. Evolution of lifestyles in Capnodiales. Studies in Mycology 95: 381–414. 32855743 10.1016/j.simyco.2020.02.004 PMC7426231

[nph18761-bib-0002] Barelli L , Behie SW , Bidochka MJ . 2019. Availability of carbon and nitrogen in soil affects *Metarhizium robertsii* root colonization and transfer of insect‐derived nitrogen. FEMS Microbiology Ecology 95: fiz144. 31504453 10.1093/femsec/fiz144

[nph18761-bib-0003] Bayman P , Otero JT . 2006. Microbial endophytes of orchid roots. In: Schulz BJE , Boyle CJC , Sieber TN , eds. Microbial root endophytes. Berlin & Heidelberg, Germany: Springer, 153–177.

[nph18761-bib-0004] Bazile V , Moran JA , Le Moguedec G , Marshall DJ , Gaume L . 2012. A carnivorous plant fed by its ant symbiont: a unique multi‐faceted nutritional mutualism. PLoS ONE 7: e36179. 22590524 10.1371/journal.pone.0036179 PMC3348942

[nph18761-bib-0005] Beattie AJ . 1985. The evolutionary ecology of ant‐plant mutualisms. Cambridge, UK: Cambridge University Press.

[nph18761-bib-0006] Behie SW , Bidochka MJ . 2014. Nutrient transfer in plant–fungal symbioses. Trends in Plant Science 19: 734–740. 25022353 10.1016/j.tplants.2014.06.007

[nph18761-bib-0007] Behie SW , Moreira CC , Sementchoukova I , Barelli L , Zelisko PM , Bidochka MJ . 2017. Carbon translocation from a plant to an insect–pathogenic endophytic fungus. Nature Communications 8: 14245. 10.1038/ncomms14245 PMC5253661 28098142

[nph18761-bib-0008] Behie SW , Zelisko PM , Bidochka MJ . 2012. Endophytic insect–parasitic fungi translocate nitrogen directly from insects to plants. Science 336: 1576–1577. 22723421 10.1126/science.1222289

[nph18761-bib-0009] Benzing DH . 1970. Foliar permeability and the absorption of minerals and organic nitrogen by certain tank bromeliads. Botanical Gazette 131: 23–31.

[nph18761-bib-0010] Benzing DH . 1990. Vascular epiphytes: general biology and related biota. Cambridge, UK: Cambridge University Press.

[nph18761-bib-0011] Benzing DH . 2000. Bromeliaceae: profile of an adaptive radiation. Cambridge, UK: Cambridge University Press.

[nph18761-bib-0012] Blatrix R , Kidyoo A , Kidyoo M , Piapukiew J , Satjarak A , Paliyavuth C , Boonchai W , McKey D . 2021. The symbiosis between Philidris ants and the ant‐plant *Dischidia major* includes fungal and algal associates. Symbiosis 83: 305–315.

[nph18761-bib-0013] Blüthgen N , Schmit‐Neuerburg V , Engwald S , Barthlott W . 2001. Ants as epiphyte gardeners: comparing the nutrient quality of ant and termite canopy substrates in a Venezuelan lowland rain forest. Journal of Tropical Ecology 17: 887–894.

[nph18761-bib-0014] Bougoure JJ , Bougoure DS , Cairney JWG , Dearnaley JDW . 2005. ITS‐RFLP and sequence analysis of endophytes from *Acianthus*, *Caladenia* and *Pterostylis* (Orchidaceae) in southeastern Queensland. Mycological Research 109: 452–460. 15912933 10.1017/s095375620500225x

[nph18761-bib-0015] Carroll G . 1988. Fungal endophytes in stems and leaves – from latent pathogen to mutualistic symbiont. Ecology 69: 2–9.

[nph18761-bib-0016] Castillo Lopez D , Sword GA . 2015. The endophytic fungal entomopathogens *Beauveria bassiana* and *Purpureocillium lilacinum* enhance the growth of cultivated cotton (*Gossypium hirsutum*) and negatively affect survival of the cotton bollworm (*Helicoverpa zea*). Biological Control 89: 53–60.

[nph18761-bib-0017] Chomicki G , Renner SS . 2015. Phylogenetics and molecular clocks reveal the repeated evolution of ant‐plants after the late Miocene in Africa and the early Miocene in Australasia and the Neotropics. New Phytologist 207: 411–424. 25616013 10.1111/nph.13271

[nph18761-bib-0018] Chomicki G , Renner SS . 2019. Farming by ants remodels nutrient uptake in epiphytes. New Phytologist 223: 2011–2023. 31236967 10.1111/nph.15855

[nph18761-bib-0019] Dearnaley JDW . 2007. Further advances in orchid mycorrhizal research. Mycorrhiza 17: 475–486. 17582535 10.1007/s00572-007-0138-1

[nph18761-bib-0020] Defossez E , Djieto‐Lordon C , McKey D , Selosse MA , Blatrix R . 2011. Plant‐ants feed their host plant, but above all a fungal symbiont to recycle nitrogen. Proceedings of the Royal Society B: Biological Sciences 278: 1419–1426. 10.1098/rspb.2010.1884 PMC3061138 20980297

[nph18761-bib-0021] Defossez E , Selosse MA , Dubois MP , Mondolot L , Faccio A , Djieto‐Lordon C , McKey D , Blatrix R . 2009. Ant‐plants and fungi: a new threeway symbiosis. New Phytologist 182: 942–949. 19383109 10.1111/j.1469-8137.2009.02793.x

[nph18761-bib-0022] Farji‐Brener AG , Werenkraut V . 2017. The effects of ant nests on soil fertility and plant performance: a meta‐analysis. Journal of Animal Ecology 86: 866–877. 28369906 10.1111/1365-2656.12672

[nph18761-bib-0023] Fischer RC , Wanek W , Richter A , Mayer V . 2003. Do ants feed plants? A ^15^N labelling study of nitrogen fluxes from ants to plants in the mutualism of *Pheidole* and *Piper* . Journal of Ecology 91: 126–134.

[nph18761-bib-0024] Gay H . 1993. Animal‐fed plants: an investigation into the uptake of ant‐derived nutrients by the far‐eastern epiphytic fern *Lecanopteris* Reinw. (Polypodiaceae). Biological Journal of the Linnean Society 50: 221–233.

[nph18761-bib-0025] Gegenbauer C , Mayer VE , Zotz G , Richter A . 2012. Uptake of ant‐derived nitrogen in the myrmecophytic orchid *Caularthron bilamellatum* . Annals of Botany 110: 757–765. 22778148 10.1093/aob/mcs140 PMC3423799

[nph18761-bib-0026] González‐Teuber M , Urzúa A , Plaza P , Bascuñán‐Godoy L . 2018. Effects of root endophytic fungi on response of *Chenopodium quinoa* to drought stress. Plant Ecology 219: 231–240.

[nph18761-bib-0027] Gostinčar C , Muggia L , Grube M . 2012. Polyextremotolerant black fungi: oligotrophism, adaptive potential, and a link to lichen symbioses. Frontiers in Microbiology 3: 390. 23162543 10.3389/fmicb.2012.00390 PMC3492852

[nph18761-bib-0028] Gotsch SG , Nadkarni N , Darby A , Glunk A , Dix M , Davidson K , Dawson TE . 2015. Life in the treetops: ecophysiological strategies of canopy epiphytes in a tropical montane cloud forest. Ecological Monographs 85: 393–412.

[nph18761-bib-0031] de Hoog GS , Hermanides‐Nijhof EJ . 1977. The black yeasts and allied Hyphomycetes. Studies in Mycology 15: 1–222.

[nph18761-bib-0032] Hoppe P , Cohen S , Meibom A . 2013. NanoSIMS: technical aspects and applications in cosmochemistry and biological geochemistry. Geostandards Geoanalytical Research 37: 111–154.

[nph18761-bib-0033] Huxley CR . 1978. The ant‐plants *Myrmecodia* and *Hydnophytum* (Rubiaceae) and the relationships between their morphology, ant occupants, physiology, and ecology. New Phytologist 80: 231–268.

[nph18761-bib-0034] Igersheim A . 1993. The character states of the Caribbean monotypic endemic *Strumpfia* (Rubiaceae). Nordic Journal of Botany 13: 545–559.

[nph18761-bib-0035] Igersheim A , Cichocki O . 1996. A simple method for microtome sectioning of prehistoric charcoal specimens, embedded in 2‐hydroxyethyl methacrylate (HEMA). Review of Palaeobotany and Palynology 92: 389–393.

[nph18761-bib-0036] Janzen DH . 1974. Epiphytic myrmecophytes in Sarawak: mutualism through the feeding of plants by ants. Biotropica 6: 237–259.

[nph18761-bib-0037] Leger RJS , Wang JB . 2020. *Metarhizium*: jack of all trades, master of many. Open Biology 10: 200307. 33292103 10.1098/rsob.200307 PMC7776561

[nph18761-bib-0038] Leroy C , Jauneau A , Martinez Y , Cabin‐Flaman A , Gibouin D , Orivel J , Séjalon‐Delmas N . 2017. Exploring fungus–plant N transfer in a tripartite ant–plant–fungus mutualism. Annals of Botany 120: 417–426. 28633407 10.1093/aob/mcx064 PMC5591417

[nph18761-bib-0039] Leroy C , Sejalon‐Delmas N , Jauneau A , Ruiz‐Gonzalez M‐X , Gryta H , Jargeat P , Corbara B , Dejean A , Orivel J . 2011. Trophic mediation by a fungus in an ant‐plant mutualism. Journal of Ecology 99: 583–590.

[nph18761-bib-0040] Looby CI , Hollenbeck EC , Treseder KK . 2020. Fungi in the canopy: how soil fungi and extracellular enzymes differ between canopy and ground soils. Ecosystems 23: 768–782.

[nph18761-bib-0041] Lucas JM , Clay NA , Kaspari M . 2018. Nutrient transfer supports a beneficial relationship between the canopy ant, *Azteca trigona*, and its host tree. Ecological Entomology 43: 621–628.

[nph18761-bib-0043] Mayer VE , Nepel M , Blatrix R , Oberhauser FB , Fiedler K , Schönenberger J , Voglmayr H . 2018. Transmission of fungal partners to incipient *Cecropia*‐tree ant colonies. PLoS ONE 13: e0192207. 29466381 10.1371/journal.pone.0192207 PMC5821464

[nph18761-bib-0044] McCormick MK , Whigham DF , O'Neill J . 2004. Mycorrhizal diversity in photosynthetic terrestrial orchids. New Phytologist 163: 425–438. 33873625 10.1111/j.1469-8137.2004.01114.x

[nph18761-bib-0045] Moghaddam Hosseyni MS , Safaie N , Soltani J , Hagh‐Doust N . 2021. Desert‐adapted fungal endophytes induce salinity and drought stress resistance in model crops. Plant Physiology and Biochemistry 160: 225–238. 33517220 10.1016/j.plaphy.2021.01.022

[nph18761-bib-0046] Moreno LF , Mayer VE , Voglmayr H , Blatrix R , Stielow B , Teixeira MM , Vicente VA , de Hoog S . 2019. Genomic analysis of ant domatia‐associated melanized fungi (Chaetothyriales, Ascomycota). Mycological Progress 18: 541–552.

[nph18761-bib-0047] Nakamura A , Kitching RL , Cao M , Creedy TJ , Fayle TM , Freiberg M , Hewitt CN , Itioka T , Koh LP , Ma K *et al*. 2017. Forests and their canopies: achievements and horizons in canopy science. Trends in Ecology & Evolution 32: 438–451. 28359572 10.1016/j.tree.2017.02.020

[nph18761-bib-0048] Nepel M , Voglmayr H , Schönenberger J , Mayer VE . 2014. High diversity and low specificity of Chaetothyrialean fungi in carton galleries in a Neotropical ant–plant association. PLoS ONE 9: e112756. 25398091 10.1371/journal.pone.0112756 PMC4232418

[nph18761-bib-0080] Peterson BJ , Fry B . 1987. Stable isotopes in ecosystem studies. Annual Review of Ecology and Systematics 18: 293–320.

[nph18761-bib-0049] Quan Y , Muggia L , Moreno LF , Wang M , Al‐Hatmi AMS , da Silva MN , Shi D , Deng S , Ahmed S , Hyde KD *et al*. 2020. A re‐evaluation of the Chaetothyriales using criteria of comparative biology. Fungal Diversity 103: 47–85.

[nph18761-bib-0050] Rains KC , Nadkarni NM , Bledsoe CS . 2003. Epiphytic and terrestrial mycorrhizas in a lower montane Costa Rican cloud forest. Mycorrhiza 13: 257–264. 14593519 10.1007/s00572-003-0224-y

[nph18761-bib-0051] Rasmussen HN . 2002. Recent developments in the study of orchid mycorrhiza. Plant and Soil 244: 149–163.

[nph18761-bib-0052] Ratnaweera PB , de Silva ED . 2017. Endophytic fungi: a remarkable source of biologically active secondary metabolites. In: Maheshwari D , Annapurna K , eds. Endophytes: crop productivity and protection. Sustainable development and biodiversity, vol. 16. Cham, Switzerland: Springer, 191–212.

[nph18761-bib-0053] Redman RS , Dunigan DD , Rodriguez RJ . 2001. Fungal symbiosis from mutualism to parasitism: who controls the outcome, host or invader? New Phytologist 151: 705–716. 33853254 10.1046/j.0028-646x.2001.00210.x

[nph18761-bib-0054] Rickson FR . 1979. Absorption of animal tissue breakdown products into a plant stem – the feeding of a plant by ants. American Journal of Botany 66: 87–90.

[nph18761-bib-0055] Rico Gray V . 1987. Schomburgkia tibicinis *Batem. (Orchidaceae) – effect of myrmecophily on reproductive fitness* . PhD dissertation, Tulane University.

[nph18761-bib-0056] Rico Gray V , Barber JT , Thien LB , Ellgaard EG , Toney JJ . 1989. An unusual animal–plant interaction: feeding of *Schomburgkia tibicinis* (Orchidaceae) by ants. American Journal of Botany 76: 603–608.

[nph18761-bib-0058] Rodriguez RJ , Henson J , Van Volkenburgh E , Hoy M , Wright L , Beckwith F , Kim Y‐O , Redman RS . 2008. Stress tolerance in plants *via* habitat‐adapted symbiosis. The ISME Journal 2: 404–416. 18256707 10.1038/ismej.2007.106

[nph18761-bib-0059] RStudio Team . 2020. RStudio: integrated development for R. Boston, MA, USA: RStudio PBC. [WWW document] URL http://www.rstudio.com/ [accessed 24 November 2022].

[nph18761-bib-0060] Saikkonen K , Faeth SH , Helander M , Sullivan TJ . 1998. Fungal endophytes: a continuum of interactions with host plants. Annual Review of Ecology and Systematics 29: 319–343.

[nph18761-bib-0061] Salazar‐Cerezo S , Martinez‐Montiel N , Cruz‐Lopez MC , Martinez‐Contreras RD . 2018. Fungal diversity and community composition of culturable fungi in *Stanhopea trigrina* cast gibberellin producers. Frontiers in Microbiology 9: Article 612. 10.3389/fmicb.2018.00612 PMC5893766 29670591

[nph18761-bib-0062] Schlick‐Steiner BC , Steiner FM , Konrad H , Seifert B , Christian E , Moder K , Stauffer C , Crozier RH . 2008. Specificity and transmission mosaic of ant nest‐wall fungi. Proceedings of the National Academy of Sciences, USA 105: 940–943. 10.1073/pnas.0708320105 PMC2242721 18195358

[nph18761-bib-0063] Solano PJ , Dejean A . 2004. Ant‐fed plants: comparison between three geophytic myrmecophytes. Biological Journal of the Linnean Society 83: 433–439.

[nph18761-bib-0064] Staedler YM , Masson D , Schönenberger J . 2013. Plant tissues in 3D *via* X‐ray tomography: simple contrasting methods allow high resolution imaging. PLoS ONE 8: e75295. 24086499 10.1371/journal.pone.0075295 PMC3785515

[nph18761-bib-0065] Suárez JP , Weiß M , Abele A , Garnica S , Oberwinkler F , Kottke I . 2006. Diverse tulasnelloid fungi form mycorrhizas with epiphytic orchids in an Andean cloud forest. Mycological Research 110: 1257–1270. 17081740 10.1016/j.mycres.2006.08.004

[nph18761-bib-0066] Sun M , Feng C‐H , Liu Z‐Y , Tian K . 2020. Evolutionary correlation of water‐related traits between different structures of *Dendrobium* plants. Botanical Studies 61: 16. 32417994 10.1186/s40529-020-00292-4 PMC7230118

[nph18761-bib-0067] Treseder KK , Davidson DW , Ehleringer JR . 1995. Absorption of ant‐provided carbon dioxide and nitrogen by a tropical epiphyte. Nature 375: 137–139.

[nph18761-bib-0068] Vasse M , Voglmayr H , Mayer VE , Gueidan C , Nepel M , Moreno L , de Hoog S , Selosse M‐A , McKey D , Blatrix R . 2017. A phylogenetic perspective on the association between ants (Hymenoptera: Formicidae) and black yeasts (Ascomycota: Chaetothyriales). Proceedings of the Royal Society B: Biological Sciences 284: 20162519. 10.1098/rspb.2016.2519 PMC5360919 28298348

[nph18761-bib-0069] Vaz ABM , Mota RC , Bomfim MRQ , Vieira MLA , Zani CL , Rosa CA , Rosa LH . 2009. Antimicrobial activity of endophytic fungi associated with Orchidaceae in Brazil. Canadian Journal of Microbiology 55: 1381–1391. 20029530 10.1139/W09-101

[nph18761-bib-0070] Voglmayr H , Jaklitsch WM . 2011. Molecular data reveal high host specificity in the phylogenetically isolated genus *Massaria* (Ascomycota, Massariaceae). Fungal Diversity 46: 133–170.

[nph18761-bib-0071] Voglmayr H , Mayer V , Maschwitz U , Moog J , Djieto‐Lordon C , Blatrix R . 2011. The diversity of ant‐associated black yeasts: insights into a newly discovered world of symbiotic interactions. Fungal Biology 115: 1077–1091. 21944219 10.1016/j.funbio.2010.11.006

[nph18761-bib-0072] Winkler U , Zotz G . 2010. ‘And then there were three’: highly efficient uptake of potassium by foliar trichomes of epiphytic bromeliads. Annals of Botany 106: 421–427. 20542886 10.1093/aob/mcq120 PMC2924823

[nph18761-bib-0073] Wu B , Cox MP . 2021. Comparative genomics reveals a core gene toolbox for lifestyle transitions in Hypocreales fungi. Environmental Microbiology 23: 3251–3264. 33939870 10.1111/1462-2920.15554 PMC8360070

[nph18761-bib-0074] Yuan Z , Chen Y , Yang Y . 2009. Diverse non‐mycorrhizal fungal endophytes inhabiting an epiphytic, medicinal orchid (*Dendrobium nobile*): estimation and characterization. World Journal of Microbiology and Biotechnology 25: 295–303.

[nph18761-bib-0075] Zotz G . 1999. What are backshoots good for? Seasonal changes in mineral, carbohydrate, and water content of different organs of the epiphytic orchid, *Dimerandra emarginata* . Annals of Botany 84: 791–798.

[nph18761-bib-0076] Zotz G , Hietz P . 2001. The physiological ecology of vascular epiphytes: current knowledge, open questions. Journal of Experimental Botany 52: 2067–2078. 11604445 10.1093/jexbot/52.364.2067

[nph18761-bib-0077] Zotz G , Hietz P , Einzmann HJR . 2021. Functional ecology of vascular epiphytes. Annual Plant Reviews Online 4: 869–905.

[nph18761-bib-0078] Zotz G , Richter A . 2006. Changes in carbohydrate and nutrient contents throughout a reproductive cycle indicate that phosphorus is a limiting nutrient in the epiphytic bromeliad, *Werauhia sanguinolenta* . Annals of Botany 97: 745–754. 16497701 10.1093/aob/mcl026 PMC2803411

[nph18761-bib-0079] Zotz G , Winkler U . 2013. Aerial roots of epiphytic orchids: the velamen radicum and its role in water and nutrient uptake. Oecologia 171: 733–741. 23292456 10.1007/s00442-012-2575-6

