# Supplementary material for: Exo‐ and endophytic fungi enable rapid transfer of nutrients from ant waste to orchid tissue
Source: New Phytol. 2023 Feb 17;238(5):2210–23. doi: 10.1111/nph.18761 (PMC10962571; doi:10.1111/nph.18761)
Supplement: Supplementary file 1 — Fig. S1 Annual growth cycle and hypothetical nutrient transfer. Fig. S2 Morphology of pseudobulb chamber inhabited by different ant species. Fig. S3 SEM images of pseudobulbs inhabited by Azteca cf velox. Fig. S4 15N enrichment in open and closed pseudobulbs. Fig. S5 Semiquantitative ToF‐SIMS image of a longitudinal section through the innermost living cells rich in endophytes displaying 12C15N− normalized to the sum of 12C15N− + 12C14N− after a labelling period of 8 d. Table S1 Sampling plots of Caularthron bilamellatum pseudobulbs and associated ant species. Table S2 M/ΔM values of negatively charged secondary ion species. Table S3 Labelling time and enrichment of pseudobulbs and leaves. Please note: Wiley is not responsible for the content or functionality of any Supporting Information supplied by the authors. Any queries (other than missing material) should be directed to the New Phytologist Central Office. [file NPH-238-2210-s001.pdf]

New Phytologist **Supporting Information**

Article title: **Exo- and endophytic fungi enable rapid transfer of nutrients from ant waste to orchid tissue**

Authors: Christian Gegenbauer, Anke Bellaire, Arno Schintlmeister, Markus C. Schmid, Markus Kubicek, Hermann Voglmayr, Gerhard Zotz, Andreas Richter, Veronika E. Mayer

Article acceptance date: 05 January 2023

The following Supporting Information is available for this article:

Fig. S1 Annual growth cycle and hypothetical nutrient transfer.

Fig. S2 Morphology of pseudobulb chamber inhabited by different ant species.

Fig. S3 SEM images of pseudobulbs inhabited by *Azteca* cf. *velox*.

Fig. S4 ^{15}N enrichment in open and closed pseudobulbs.

Fig. S5 Semi-quantitative ToF-SIMS image of longitudinal section through the innermost living cells rich in endophytes displaying $^{12}C^{15}N^-$ normalized to the sum of $^{12}C^{15}N^-$ + $^{12}C^{14}N^-$ after a labelling period of 8 days.

Table S1 Sampling plots of Caularthon bilamellatum pseudobulbs and associated ant species**.**

Table S2 M/ΔM values of negatively charged secondary ion species.

Table S3 Labelling time and enrichment of pseudobulbs and leaves.

Fig. S1 Annual growth cycle and hypothetical nutrient transfer in the myrmecophytic orchid *Caularthron bilamellatum*. Each year one generation of pseudobulbs (pb) is produced by sympodial growth along a creeping rhizome. Pbs only flower once but remain alive for several years. Red arrows indicate possible direction of nutrient flux within the plant, from storage tissue to growing organs. Blue arrows indicate water uptake. Ants inhabit hollow, mature pbs (brown) and leave detritus in the apical regions (black). Fungal endophytes can be found in the living tissue close to the pb chamber (white). (1) New growth begins in the late dry, early rainy season

in April–May. New pbs (a) and roots are produced to absorb and store water and nutrients acting as nutrient sink while mature pbs and their leaves (b) as well as leafless, older pbs from the previous seasons (c) act as nutrient source. (2) Pb and root growth is completed in the late rainy season. Large amounts of water are stored in the gel-like tissue in the central parenchyma tissue of growing pbs and in the newly formed succulent leaves. The growing pbs and their emerging leaves and inflorescences also act as nutrient sink (d) while old pbs and roots continue to act as nutrient source (e, f). Labelling experiments were carried out in this phase. (3) Flowering starts at the onset of the dry season in January. The now mature pb slowly desiccates during the dry season to compensate for the lack of precipitation. The hollow chamber as nesting space for ants is formed, a slit is opening at the base (g). Plant and root growth stop. Old growth and the now mature pb act as nutrient source for flower production and as a water source (h, i). (4) Seed-pods are produced by the now matured pb during dry season from Feb-Apr. acting as nutrient sink. Increased ant presence leads to formation of detritus and colonisation by fungal endophytes in these freshly matured pbs, creating a new nutrient source (j). The mature and old pbs desiccate shedding old leaves, releasing water and nutrients for seed production and in preparation for the formation of new growth at the onset of the next rainy season in early summer (k,l).

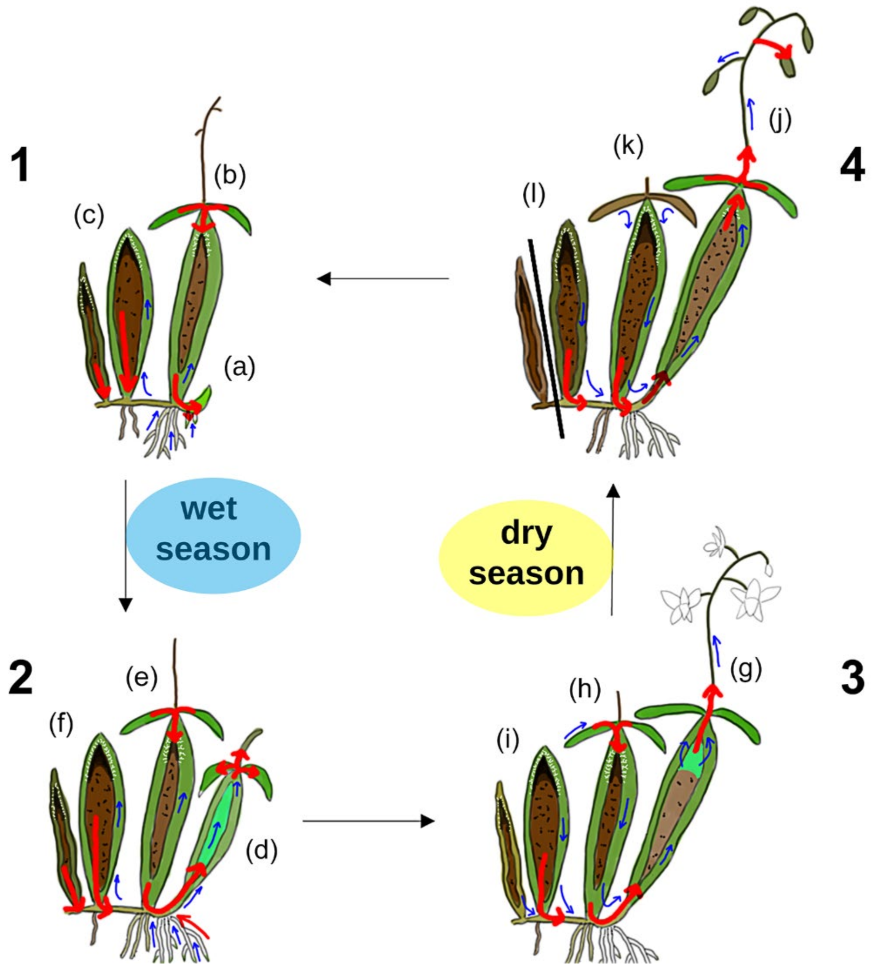

Fig. S2 Morphology of the pseudobulb (pb) chamber in *Caularthron bilamellatum* inhabited by different ant species. (a) Closed pb, having failed to form a natural opening, free of ants and microorganisms. The inner surface is light-coloured and smooth. (b) Uninhabited pb showing signs of former ant presence. The desiccated parenchyma tissue has been removed. The surface is darkened from melanized hyphae but with little detritus. (c) Pb inhabited by *Camponotus atriceps*, filled with mud-like detritus and insect remains. (d) Pb inhabited by *Pheidole* sp. retaining a light colour similar to the closed pbs but with a rough surface shaped by ants. (e) Pb inhabited by *Azteca* cf. *velox* with ant carton of reddish-brown colour. Dead tissue has been removed and some detritus was found in the apical region. (f) Pb inhabited by a larger *Azteca* species. Dark detritus with only little difference to *A.* cf *velox* is found in the apical region of the pb. (g) Typical mature pb inhabited by *Azteca* cf. *velox*. The inner surface is relatively

smooth except for the apical region where dark detritus accumulates. Ants enter through a small slit at the base, which forms naturally during pb maturation.

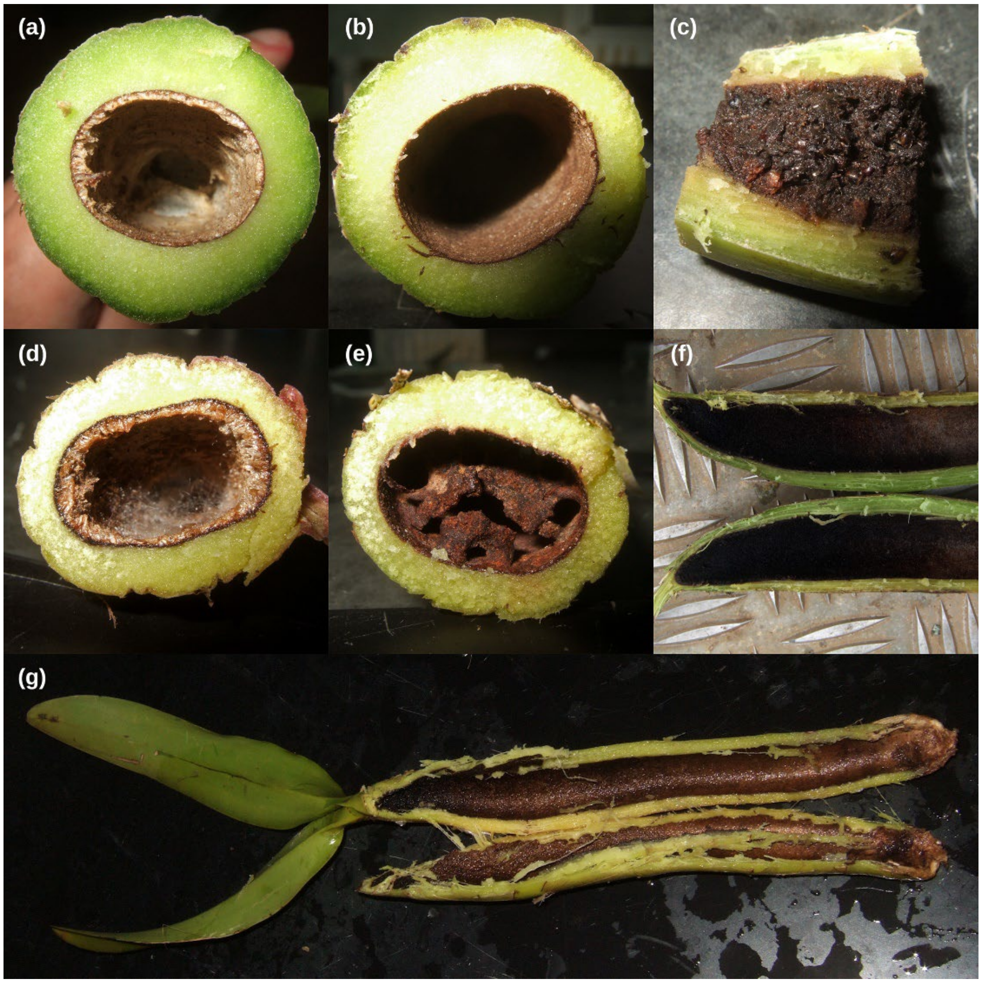

Fig. S3 Scanning electron microscopy images of *Caularthron bilamellatu*m pseudobulbs (pb) inhabited by *Azteca* cf. *velox*. (a–c) Thin fungal hyphae in living cells close to the hollow pb chamber. (a) Hyphae growing within cells and along cell walls. Note the abundance of pits. (b) Hyphae crossing from one cell to another penetrating the cell wall. (c) Hyphae growing between cell walls. (d) Thick distinctly septate hyphae (BF = black fungi) and thin hyphae (Hyp = Hypocreales) growing in the detritus layer covering the pb chamber.

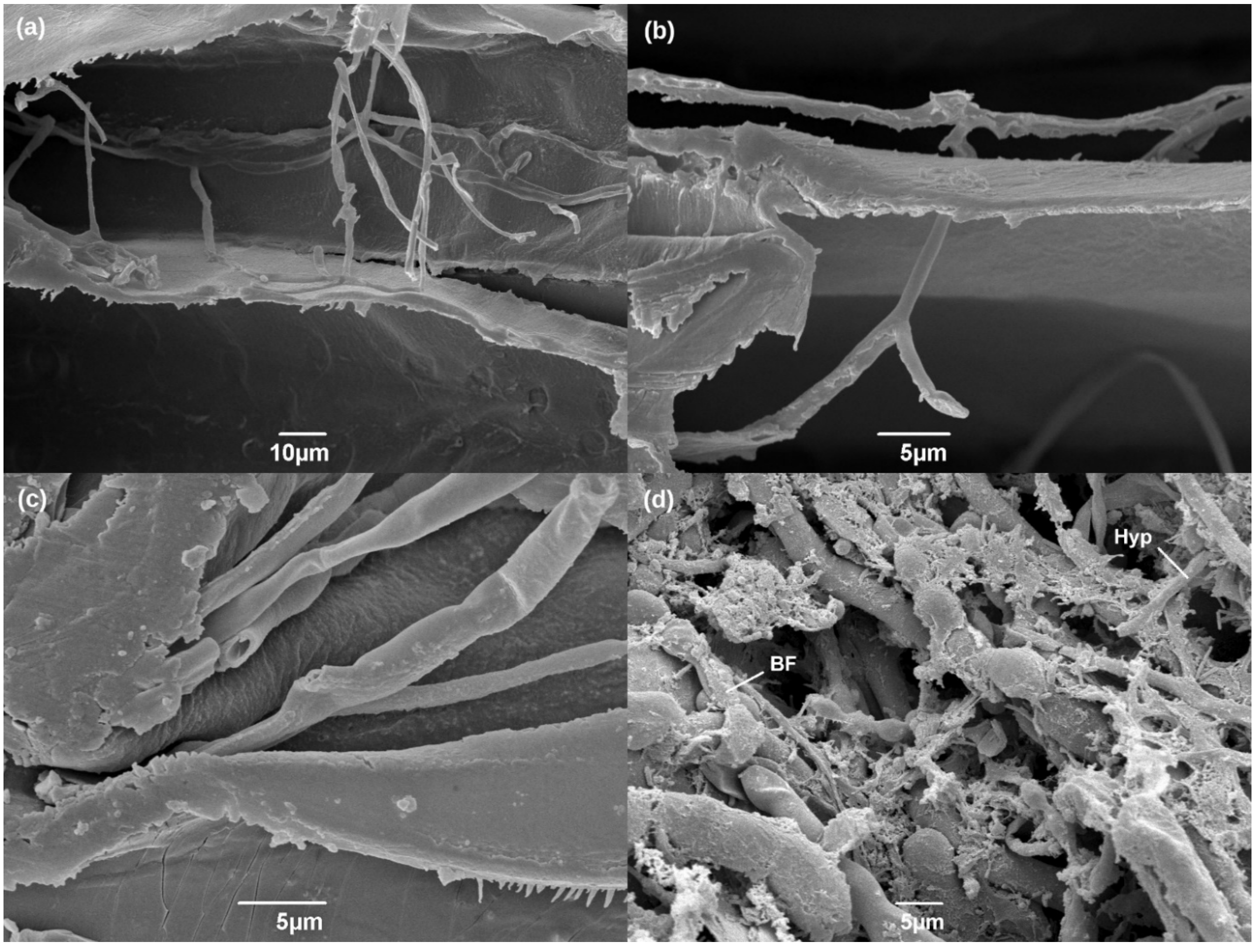

Fig. S4 ^{15}N enrichment (in atom percent excess) from *Caularthron bilamellatum* pseudobulbs which were inhabited by ants after slit formation during desiccation (open, n=40) vs. closed pbs without slit and free of ants and microorganisms (closed, n=2). No slit formation is very rare therefore the sample size for "closed" is extremely small. Box-plots show the median as horizontal line and interquartile ranges as coloured box, whiskers the minimum and maximum. Letters indicate significant differences between groups (GLM *estimate* 8.88, *t-value* 2.6, $df = 40$, $p = 0.014$).

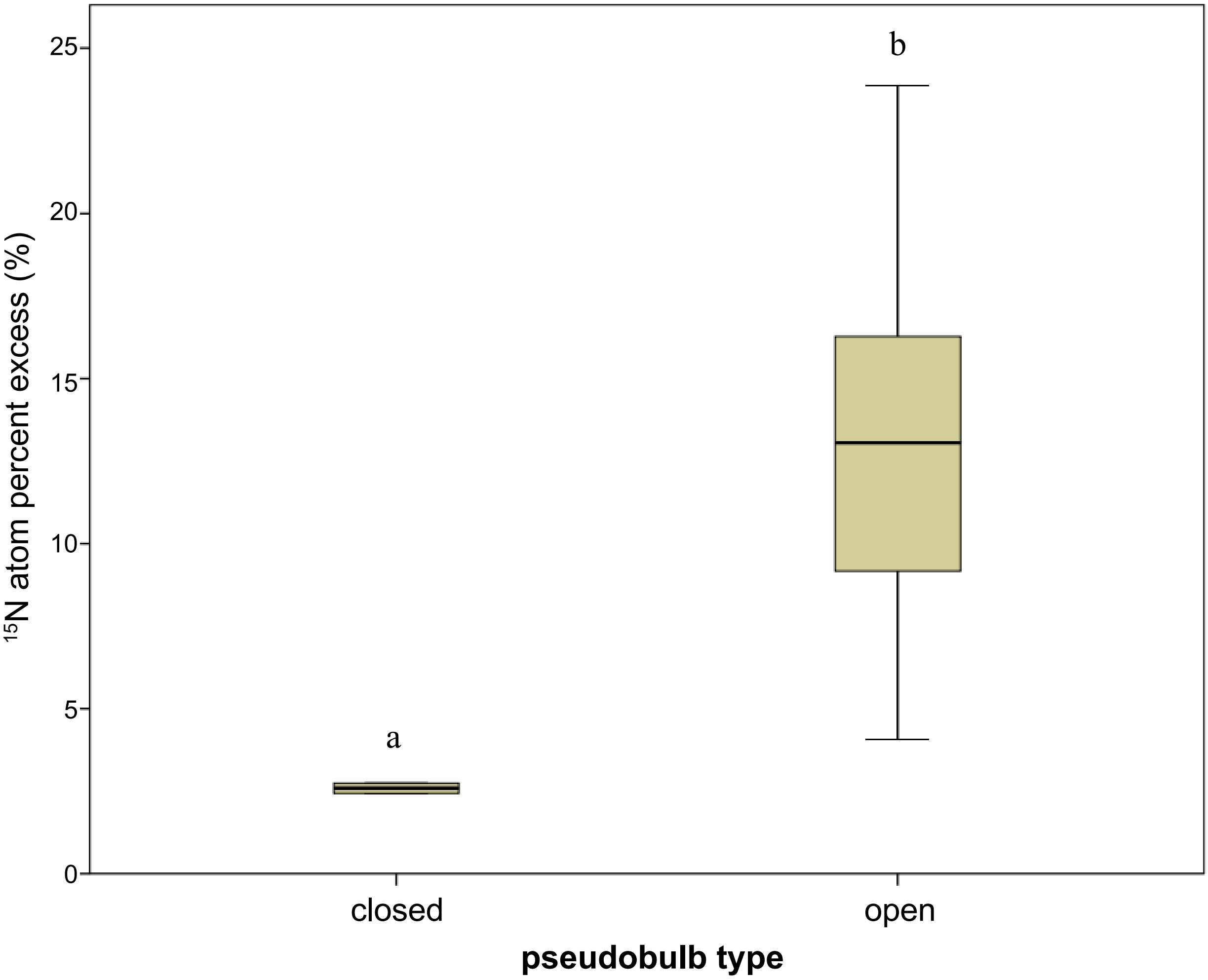

Fig. S5 Semi-quantitative ToF-SIMS image of longitudinal section through the innermost living cells rich in endophytes displaying $^{12}C^{15}N^-$ normalized to the sum of $^{12}C^{15}N^-$ + $^{12}C^{14}N^-$ after a labelling period of 8 days. The false-colour scale displays at% ^{15}N ranging from 0 (black) to 100% (white). Fungal hyphae are displayed as turquoise to yellow dots and exhibited massive enrichment up to 60 at% ^{15}N. A corresponding light microscopy image of the area surveyed by ToF-SIMS is shown on the right. All strongly labelled structures can be correlated to fungal hyphae.

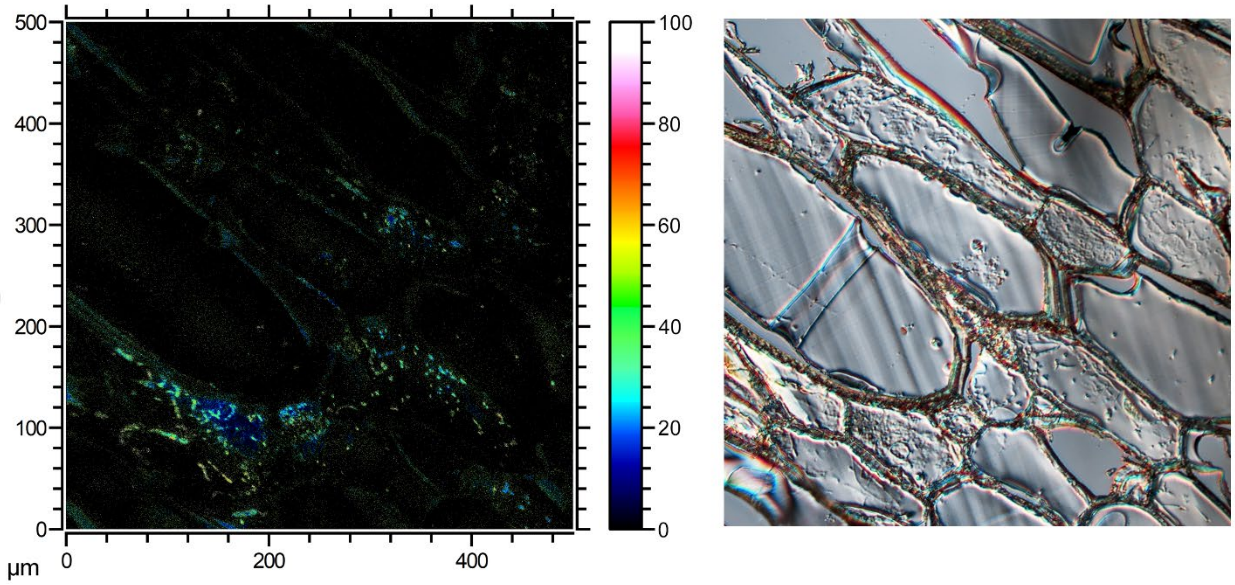

Table S1 Sampling plots of Caularthon bilamellatum (Orchidaceae) pseudobulbs (pbs) and associated ant species at Barro Colorado Island, Panama. (CL) Closed hollow pbs free of ants and microorganisms on the inside; (AB) signs of previous ant presence in pbs but devoid of ants upon sampling time; (CAM) Camponotus atriceps (Formicinae); (PHE) Pheidole sp. (Myrmicinae); (AZA) Azteca sp.; (AZ1, AZ2) Azteca cf. velox (Dolichoderinae). The amount of detritus and presence of two morphologically distinct fungi (hyphal type 1: isolates from black fungi Capnodiales, Chaetothyriales, Mycosphaerellales, and hyphal type 2: Hypocreales) on the surface of the hollow pb chamber is given as: (-) completely absent, (+) very low, (++) medium, (+++) very high. Corresponding images are shown in Supporting Information Fig. S2.

Plot designation	CL	AB	CAM	PHE	AZA	AZ1	AZ2
**Ant species inhabiting pseudobulbs**	closed ant free	abandoned	*Camponotus atriceps*	*Pheidole* sp.	*Azteca* sp.	*Azteca* cf. *velox*	*Azteca* cf. *velox*
**Hyphal type 1**	-	++	+++	+	++	++	++
**Hyphal type 2**	-	+	+	++	++	++	++
**Detritus**	-	++	+++	+	++	++	++
**Pseudobulb chamber surface**	light brown smooth	dark brown rough	dark brown detritus layer	light brown rough	dark brown rough	dark brown rough	dark brown rough
**Image (Fig. S2)**	A	B	C	D	F	E,G	E,G
**IRMS pseudobulb sample size**	n=2	n=7	n=4	n=7	n=8	n=7	n=7
**IRMS leaf sample size**	n=0	n=6	n=6	n=6	n=8	n=6	n=6

Table S2 M/ΔM values of negatively charged secondary ion species with nominal masses 26 and 27, relative to 12C14N- and 12C15N-. The displayed numbers indicate the minimum required mass resolving power (MRP) essential for detection of the two targeted ion species without mass interferences.

Secondary ion species	$(M/\Delta M)_{rel.}$
$^{26}Mg^-$	1269
**$^{12}C^{14}N^-$**	-
$^{13}C_2^-$	7153
$^{10}B^{16}O^-$	5442
$^{12}C^{13}C^1H^-$	3208
$^{12}C_2{}^2H^-$	2358
$^{12}C_2{}^1H_2^-$	2068
$^{13}C^{11}B^1H_2^-$	1031
$^{12}C^{11}B^1H_3^-$	876
$^{27}Al^-$	1454
**$^{12}C^{15}N^-$**	-
$^{11}B^{16}O^-$	6568
$^{13}C^{14}N^-$	4273
$^{12}C^{14}N^1H^-$	2503
$^{13}C_2{}^1H^-$	1873
$^{12}C^{13}C^1H_2^-$	1430
$^{12}C_2{}^1H_3^-$	1156

Table S3 Labelling time and enrichment (in atom percent excess APE% ± standard error) of pseudobulbs and leaves of Caularthron bilamellatum. The incubation time had an effect on 15N enrichment of the pseudobulb tissue (APE% 15N: GLM estimate 1.53, t-value 2.1, df = 40, $p = 0.043$) and the corresponding leaves (GLM estimate 0.009, t-value 2.1, df = 35, $p = 0.04$) with a significantly higher enrichment at day 8 compared to day 1 ($p = 0.023$, Kruskal-Wallis rank sum test with a Dunn post hoc analysis).

**Labeling time (in days)**	**1**	**2**	**4**	**8**
**APE [%] pseudobulbs**	8.5 ±1.3	10.4 ±1.4	13.1 ±1.6	13.3 ±1.2
**APE [%] leaves**	0.017 ±0.003	0.018 ±0.004	0.025 ±0.008	0.044 ±0.016
